# Platelet-rich fibrin as an autologous biomaterial for bone regeneration: mechanisms, applications, optimization

**DOI:** 10.3389/fbioe.2024.1286035

**Published:** 2024-04-16

**Authors:** Kewen Jia, Jiaqian You, Yuemeng Zhu, Minghui Li, Sheng Chen, Sicong Ren, Siyu Chen, Jingqi Zhang, Hanchi Wang, Yanmin Zhou

**Affiliations:** Jilin Provincial Key Laboratory of Tooth Development and Bone Remodeling, Hospital of Stomatology, Jilin University, Changchun, Jilin, China

**Keywords:** platelet-rich derivatives, platelet-rich fibrin, bone regeneration, bone tissue engineering, dentistry

## Abstract

Platelet-rich fibrin, a classical autologous-derived bioactive material, consists of a fibrin scaffold and its internal loading of growth factors, platelets, and leukocytes, with the gradual degradation of the fibrin scaffold and the slow release of physiological doses of growth factors. PRF promotes vascular regeneration, promotes the proliferation and migration of osteoblast-related cells such as mesenchymal cells, osteoblasts, and osteoclasts while having certain immunomodulatory and anti-bacterial effects. PRF has excellent osteogenic potential and has been widely used in the field of bone tissue engineering and dentistry. However, there are still some limitations of PRF, and the improvement of its biological properties is one of the most important issues to be solved. Therefore, it is often combined with bone tissue engineering scaffolds to enhance its mechanical properties and delay its degradation. In this paper, we present a systematic review of the development of platelet-rich derivatives, the structure and biological properties of PRF, osteogenic mechanisms, applications, and optimization to broaden their clinical applications and provide guidance for their clinical translation.

## 1 Introduction

Tumors, post-traumatic fractures, and osteomyelitis following septic bacterial infections are common causes of bone defects (Zhao et al., 2019; Zhu et al., 2021; Jung et al., 2022). With the concerted efforts of researchers and clinicians, introducing bone graft substitutes developed through bone tissue engineering to repair localized bone defects is gradually becoming an effective treatment. During bone regeneration, the bone graft substitute needs to form a functional connection with the living bone, which usually requires the following characteristics: (1) good biocompatibility of the material; (2) osteoconduction of the implant material (Albrektsson and Johansson, 2001); and (3) osteoinduction of the implant material (Fitzpatrick et al., 2021). Among these, osteoconduction is the property that allows the bone to grow on the surface of the bone substitute material (BSM) or to grow downward into the voids or channels of the material (Weber, 2019). In bone healing, the extracellular matrix (ECM) component that exerts an osteoconductive role allows differentiated osteoblasts to adhere, migrate, and proliferate at the site of injury while its interconnected voids facilitate adequate blood vessel formation (Malagon-Escandon et al., 2021). Mesenchymal stem cells (MSCs) present in bone and its surrounding tissues that are essential for bone healing or implant fixation, but are less differentiated, can be recruited to form bone progenitor cells and develop into differentiated osteoblasts over time with appropriate stimulation (common inducing agents, such as growth factors), which is a process known as osteoinduction (Albrektsson and Johansson, 2001; Vermeulen et al., 2022).

Many bone graft substitutes are used for bone tissue engineering, including autografts, allografts, xenografts, and synthetic bone substitution material (Rolvien et al., 2018), among which autologous bone grafts are the gold standard of bone graft materials. They contain MSCs, osteoblasts, and growth factors required for bone regeneration, providing good osteoinductive properties (Zhang et al., 2021). However, autografts have limited clinical application due to their limited source, the need to open a secondary surgical area, and their susceptibility to postoperative complications (Pepelassi et al., 2019). Allografts, xenografts, and inert bone graft materials commonly used in bone tissue engineering, such as tricalcium phosphate (Taktak et al., 2018), nano-hydroxyapatite (Jeong et al., 2022), and porous barium titanate scaffolds (Liu et al., 2020), although they can be used as support structures for bone regeneration, their ability to regenerate skeletons is restricted due to the absence of autologous cells and growth factors. Therefore, bone morphogenetic protein-2 (BMP-2) (Chen X. et al., 2021), nerve growth factor, vascular endothelial growth factor (VEGF), and interferon-γ (Li T. et al., 2018) are usually used in bone tissue engineering to functionalize bone graft materials to enhance their ability to induce osteogenesis. It is imperative to address the limitations of exogenous growth factors such as BMP-2, which hinder their applicability in clinical settings due to their high cost, severe side effects, and improper dosages (Gillman and Jayasuriya, 2021). Thus, developing a new tissue engineering material with a porous scaffold structure is crucial. This material should offer sustained release of autologous growth factors at physiologically relevant doses to support bone regeneration.

Platelet-rich fibrin (PRF), as an autologous-derived bioactive material, consists of a porous fibrin scaffold, and its internal loading of growth factors, leukocytes, and platelets, which is free of immune rejection and simple and inexpensive to prepare, has been widely used in bone tissue engineering and dentistry (Ozsagir et al., 2020; Liu et al., 2021; Benalcazar Jalkh et al., 2022). The classical platelet derivative PRF has unique advantages over other generations of platelet-rich derivatives: firstly, as opposed to PRP, PRF is derived exclusively from autologous blood without the addition of any biologics, which avoids delayed wound healing caused by anticoagulants (Dohan Ehrenfest et al., 2009b; Mourao et al., 2015); the naturally agglutinated PRF forms a highly elastic fibrin scaffold, and a large amount of growth factors are gradually released with the degradation of the fibrin scaffold, which prolongs their action time, making it more suitable for long-term tissue regeneration process (Owen and Campbell, 1999; Thorwarth et al., 2005; Kobayashi et al., 2016; Cheng et al., 2018). Secondly, as opposed to CGF, compared with the highly variable centrifugation method of CGF, the preparation of PRF is simpler and the quality of PRF is more controllable. What’s more, physiological doses of growth factors and platelets in PRF may be more favorable for tissue regeneration compared to CGF, where supraphysiological concentrations of growth factors and platelets may lead to tissue edema and unfavorable cellular responses (Weibrich et al., 2004; Chen J. et al., 2021). Thirdly, although platelet-derived extracellular vesicles have attracted great attention as new stars of platelet derivatives, the preparation, isolation and purification of platelet-derived extracellular vesicles are difficult and the possibility of clinical translation, safety and side effects need to be further studied (Antich-Rossello et al., 2021). On the contrary, PRF, as a classical regenerative material, has been widely used in oral bone regeneration, such as implantology, periodontology, and orthodontics. In addition, in bone tissue engineering, PRF is loaded with physiological doses of growth factors and cells that heighten osteoinductive properties and enhance the biocompatibility of bone graft materials. Also, its fibrin scaffold can be used as a drug delivery system. However, it still has some limitations, so how to enhance the mechanical properties of PRF and delay its degradation has become one of the urgent clinical problems to be solved. This article systematically reviews the development of platelet-rich derivatives, the structure and biological properties of PRF, the osteogenic mechanism and its application and optimization to guide its application in bone tissue engineering and clinical translation.

## 2 The evolution and characteristics of platelet-rich derivatives

**FIGURE 1:**
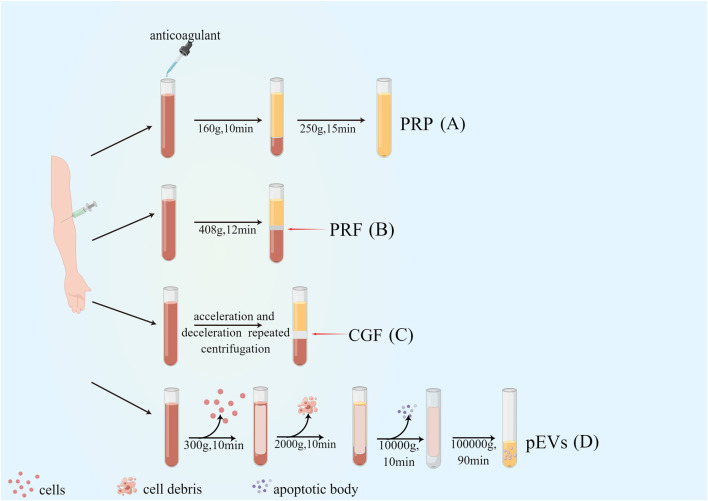
Preparation and evolution of platelet-rich derivatives. **(A)** First-generation platelet-rich derivatives: platelet-rich plasma (PRP); **(B)** Second-generation platelet-rich derivatives: Platelet-rich fibrin (PRF); **(C)** Third-generation platelet-rich derivatives: concentrated growth factor (CGF); **(D)** Novel platelet-rich derivatives: platelet-derived extracellular vesicles (pEVs).

Significant progress has been made in platelet-rich derivatives since their first preparation in 1980s. From the first generation of platelet-rich derivatives by double centrifugation to the combination with tissue engineering scaffolds, the preparation methods and composition have been optimized and improved to different degrees, facilitating the biological behavior and broadening the scope of their clinical applications. The evolution of platelet derivatives and their preparations are shown in Figure 1 and Table 1.

**TABLE 1:** The evolution of platelet derivatives and their preparations.

Platelet-rich derivatives		Centrifuge process (centrifugal force or revolution per minute and time of centrifugation)	Special characteristics
first-generation platelet-rich derivatives: PRP	L-PRP	firstly, 250×g for 10 min, secondly 250×g for 10 min. (Jia et al., 2018)	the addition of an anticoagulant was required before centrifugation
	P-PRP	firstly, 160×g for 10 min, secondly 250×g for 15 min. (Jia et al., 2018)	
second-generation platelet-rich derivatives: PRF	L-PRF	400×g for 12 min (Ratajczak et al., 2018)	none
	H-PRF	700×g for 8 min (Feng et al., 2020)	horizontal centrifugation
	T-PRF	2,700 rpm for 12 min (Ercan et al., 2022)	titanium tubes
	i-PRF	60×g for 3 min (Ozsagir et al., 2020)	none
	A-PRF	100×g for 14 min (Kobayashi et al., 2016)	none
	Ly-PRF	400×g for 10 min (Ngah et al., 2021)	to prepare Ly-PRF, intact fresh PRF was frozen and stored at −80°C for 30 min before being freeze-dried overnight at −51°C to produce Ly-PRF.
third-generation platelet-rich derivatives: concentrated growth factor (CGF)		accelerate for 30 s, then 2,700 rpm for 2 min, 2,400 rpm for 4 min, 2,700 rpm for 4 min, 3,000 rpm for 3 min, finally, decelerate for 36 s and stop	acceleration and deceleration repeated centrifugation
platelet-derived extracellular vesicles (pEVs)		300–2,000×g for 20 min, 5,000–10000×g for 10 min, and 1,000,00× g for 1–3 h	none

### 2.1 First-generation platelet-rich derivatives: platelet-rich plasma (PRP)

PRP was first successfully produced from human blood by centrifugation in 1980s (Xu et al., 2017). PRP is categorized into pure PRP (pure PRP, P-PRP), leukocyte and platelet-rich plasma (leukocyte and PRP, L-PRP), depending on the method of preparation and its contents. Yin et al. prepared P-PRP by the way that after centrifugation at 160×g for 10 min, the blood was divided into erythrocyte layer, haematocrit layer and plasma layer from bottom to top, and the plasma layer was aspirated and centrifuged again at 250×g for 15 min, the supernatant was discarded, and a small amount of serum was left to mix with the bottom precipitate, the result was P-PRP, and this protocol allowed continuous capture and concentration of platelets and growth factors, effectively preserving platelet function (Yin et al., 2017). Based on that, Julia Etulain et al. further improved the preparation protocol of PRP by dilution, low temperature, and addition of cryoprecipitate to improve PRP’s vascular regeneration and tissue formation properties. According to the findings, it is clear that the altered PRP was highly influential in stimulating platelet activation and the release of α-granules, and the modified PRP could further induce the multiplication and migration of human microvascular endothelial cells-1 and promote angiogenesis (Etulain et al., 2018; Smith, 2022).

### 2.2 Second-generation platelet-rich derivatives: PRF

In 2001, PRF, a new platelet concentrate with a high number of leukocytes, was developed by Choukroun et al. It is also referred to as leukocyte and platelet-rich fibrin (L-PRF) (Dohan Ehrenfest et al., 2009b). In recent years, the improvement of the original Choukroun’s L-PRF preparation method has enabled the development of several PRF derivatives, such as PRF prepared by horizontal centrifugation (horizontal PRF, H-PRF), PRF prepared by titanium centrifuge tubes (Titanium PRF, T-PRF), advanced PRF (A-PRF) and injectable PRF (i-PRF). The primary goals of these improvements were (1) to prepare a thicker, better cross-linked, and mechanically more robust fibrin matrix, (2) to improve the content and release of growth factors, and (3) to exclude (or include) more leukocytes (Aizawa et al., 2020). Compared with L-PRF, H-PRF has a smoother cell layer distribution and more uniform blood cell separation (Fujioka-Kobayashi et al., 2021), and T-PRF has a similar basic structure to L-PRF, with no significant differences in platelet and leukocyte content; however, its fibrin arrangement is thicker and denser (Tunali et al., 2014). To broaden the application of PRF in different tissue defects *in vivo*, Hugo Almeida Varela et al. prepared i-PRF based on the concept of low-speed centrifugation at 700 rpm for 3 min (Varela et al., 2019), which is more beneficial for the repair of irregular bone defects and acute wound healing due to its injectable nature (Farshidfar et al., 2022). In order to retain the biological activity of growth factors, freshly prepared PRF must be used immediately and the poor storage of membrane or gel PRF limits its application in clinical therapy; therefore, lyophilized platelet-rich fibrin (Ly-PRF) is proposed to be prepared by lyophilization or freeze-drying methods to address the short clinical half-life of current fresh platelet concentrates (Andia et al., 2020). Moreover, the lyophilization method could improve the biological properties of PRF, mainly including the following: firstly, scanning electron microscopy showed that the pores of the fibrin network in Ly-PRF were larger and richer; secondly, the larger diameter of fibrin in Ly-PRF might enable the sustained slow release of growth factors; thirdly, Ly-PRF exhibits a relatively rough and irregular surface that is more favorable for osteoblast adhesion, growth, and differentiation (Ngah et al., 2021)**.** In addition, changing the centrifugal force and centrifugal time during PRF preparation can improve the biological properties of PRF. Also compared to high-speed centrifugation, PRF prepared by low-speed centrifugation has the following advantages: (1) higher platelet content and more uniform distribution, (2) higher concentration of growth factors secreted within 10 days, (3) smaller volume and larger fibrin voids. (4) better ability to promote tissue regeneration (Miron et al., 2020). For example, PRF (PRF-low) prepared by low-speed centrifugation (44 g) had a more remarkable ability to promote vascular regeneration (Herrera-Vizcaino et al., 2019).

### 2.3 Third-generation platelet-rich derivatives: concentrated growth factor (CGF)

In contrast to the uniform centrifugation of the first and second generation platelet-rich derivatives, Rodella et al. successfully prepared the third generation platelet-rich derivative, CGF, by repetitive centrifugation using acceleration and deceleration as follows: accelerate for 30 s, centrifuge at 2,700 rpm for 2 min, 2,400 rpm for 4 min, 2,700 rpm for 4 min, 3,000 rpm for 3 min, decelerate for 36 s and stop (Rodella et al., 2011). Due to the specificity of its preparation process, CGF contains a denser collagen matrix with higher tensile strength and more cytokines (Rodella et al., 2011; Aizawa et al., 2020; Lee et al., 2020), which can effectively promote local bone tissue/soft tissue regeneration as an emerging tissue regeneration material.

### 2.4 Platelet-derived extracellular vesicles (pEVs)

Extracellular vesicles (EVs) are membrane vesicles released from the cytoplasmic membrane (microvesicles or microparticles) or intracellular body compartments (exosomes) of cells, as described by the International Society for Extracellular Vesicles (ISEV), which is a collective term covering various subtypes of cell-released membrane structures, also known as exosomes, microvesicles, microparticles, exosomes, cancer vesicles, and apoptotic vesicles, among others (Lotvall et al., 2014; Sedgwick and D'Souza-Schorey, 2018; Thery et al., 2018). pEVs are nanoparticles derived from activated platelets and contain growth factors, procoagulant, anti-inflammatory, pro-angiogenic factors, nucleic acids (mRNA and miRNA), and mitochondria, which can also be referred to as platelet-derived exosomes (PLT-Exos) (Boilard, 2018; Marcoux et al., 2019). In regenerative medicine, centrifugation remains the most commonly employed method for preparing pEVs. A study by Rafal Szatanek et al. proved the successful preparation of pEVs through differential centrifugation at a temperature of 4°C. The first step involved centrifugation at 300–2,000 g for 20 min to remove cells, cell debris, and apoptotic vesicles. This was followed by a centrifugation at 5,000–10000 g for 10 min to segregate larger EVs and protein pellets. Finally, a higher force of 100000g was employed for 1–3 h to isolate the exosomes from the supernatant (Szatanek et al., 2015). As platelet concentrates and potential effectors of platelets themselves, pEVs play essential roles in coagulation, osteogenesis, angiogenesis (Sun et al., 2019; Bordin et al., 2022), and periodontal regeneration (Antich-Rossello et al., 2022). It has been shown that pEVs can promote osteogenic differentiation of human umbilical cord-derived mesenchymal stromal cells by upregulating the expression of Runt-related transcription factor 2/SOX9 (Runx2/SOX9) as well as Alkaline phosphatase (ALP) (Antich-Rossello et al., 2020) and in addition, bone-targeted delivery of platelet lysate exosomes may improve glucocorticoid-induced osteoporosis by enhancing bone vascular coupling (Zheng et al., 2022).

## 3 Structure and biological properties of PRF

**FIGURE 2:**
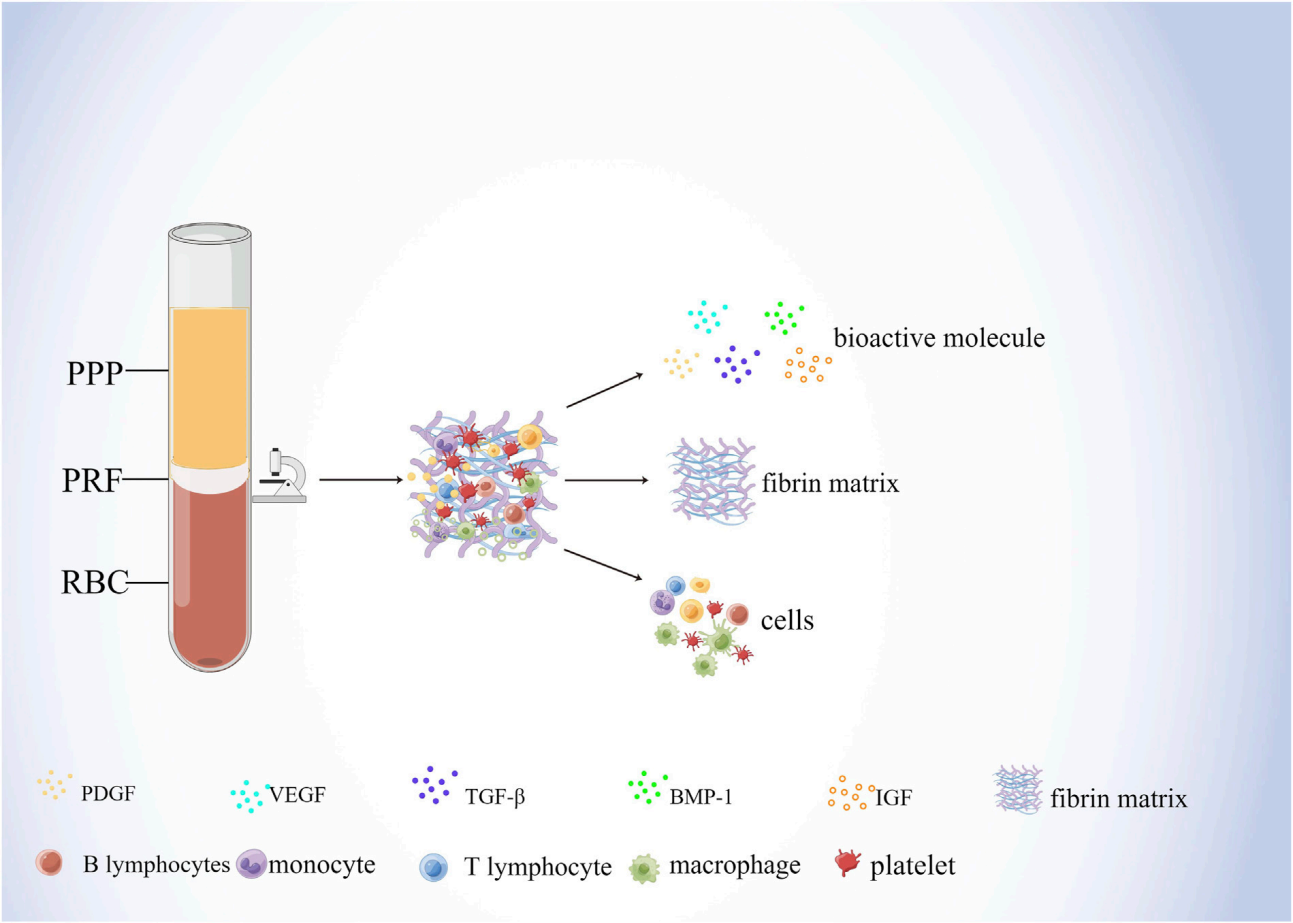
Components of PRF. Whole blood divides into three structural layers after centrifugation, namely the superficial platelet-poor plasma (PPP), the intermediate platelet-rich fibrin (PRF), and the basal layer of red blood cells (RBCs) The composition of the PRF includes bioactive molecules, fibrin matrix, and cells. Among them, bioactive molecules mainly include PDGF, VEGF, TGF-β, BMP-1, and IGF. Cells mainly include lymphocytes, macrophages, and platelets, etc.

As a biological scaffold and growth factor reservoir for tissue regeneration, PRF is a solid 3D fibrin membrane containing a dense fibrin matrix abundant in platelets and growth factors, made mainly from whole blood without anticoagulants (Kardos et al., 2018; Mazzotta et al., 2021). Whole blood divides into three structural layers after centrifugation, namely the superficial platelet-poor plasma (PPP), the intermediate platelet-rich fibrin (L-PRF), and the basal layer of red blood cells (RBCs) (Figure 2) (Kazemi et al., 2017; Kardos et al., 2018). Studies have shown that centrifugation of a 9 mL blood sample produces PRF with about 1 mL fibrin volume, which is enriched with about 97% platelets and more than 50% leukocytes in the blood (Dohan Ehrenfest et al., 2010). H&E staining showed that two distinct regions, the platelet and fibrin regions, were observed in fresh PRF; in the platelet region, high concentrations of dark purple platelets were seen; in the fibrin region, various blood cells were seen with cross-linked pink reticular structures (Kang et al., 2011). The microstructure of PRF indicates that it physically and biologically satisfies the essential properties of an ideal scaffold and that various blood cells can reside in PRF and secrete cytokines over time to promote soft and hard tissue regeneration (Madurantakam et al., 2015)**.**

Fibrin and fibronectin, the main components of PRF fibronectin scaffolds, play overlapping roles in blood coagulation, cell-matrix interactions and wound healing, initiating hemostasis and providing an initial matrix that can be used for cell adhesion, migration, proliferation, and differentiation (Noori et al., 2017) In addition, fibronectin plays an important role in blood vessel formation and bone regeneration. Interaction of fibronectin beta15-42 molecule with calmodulin, a receptor for vascular endothelial cells, promotes the formation of capillaries, and its activity is enhanced by binding to growth factors such as fibroblast growth factor-2 (FGF-2) and vascular endothelial growth factor (Mosesson, 2005). Moreover, fibrin enhances their osteogenic differentiation and promotes the expression of osteocalcin in human bone mesenchymal stem cells (BMSCs) (Lohse et al., 2012). Furthermore, fibrinogen binds to surface integrins and mediates RUNX2 expression through the SMAD1/5/8 signaling pathway to induce osteogenic differentiation of human embryonic stem cells (hESC) and induced pluripotent stem cells (iPSC) (Kidwai et al., 2016). In addition, the presence of fibrin and fibrinogen as the main components of the PRF scaffold may reduce the sensitivity of endogenous peptide growth factor to protein hydrolases, weaken the degradation of protein hydrolases and ensure the sustained release of growth factor (Lundquist et al., 2008), which prolongs the duration of action of PRF to some extent.

**FIGURE 3:**
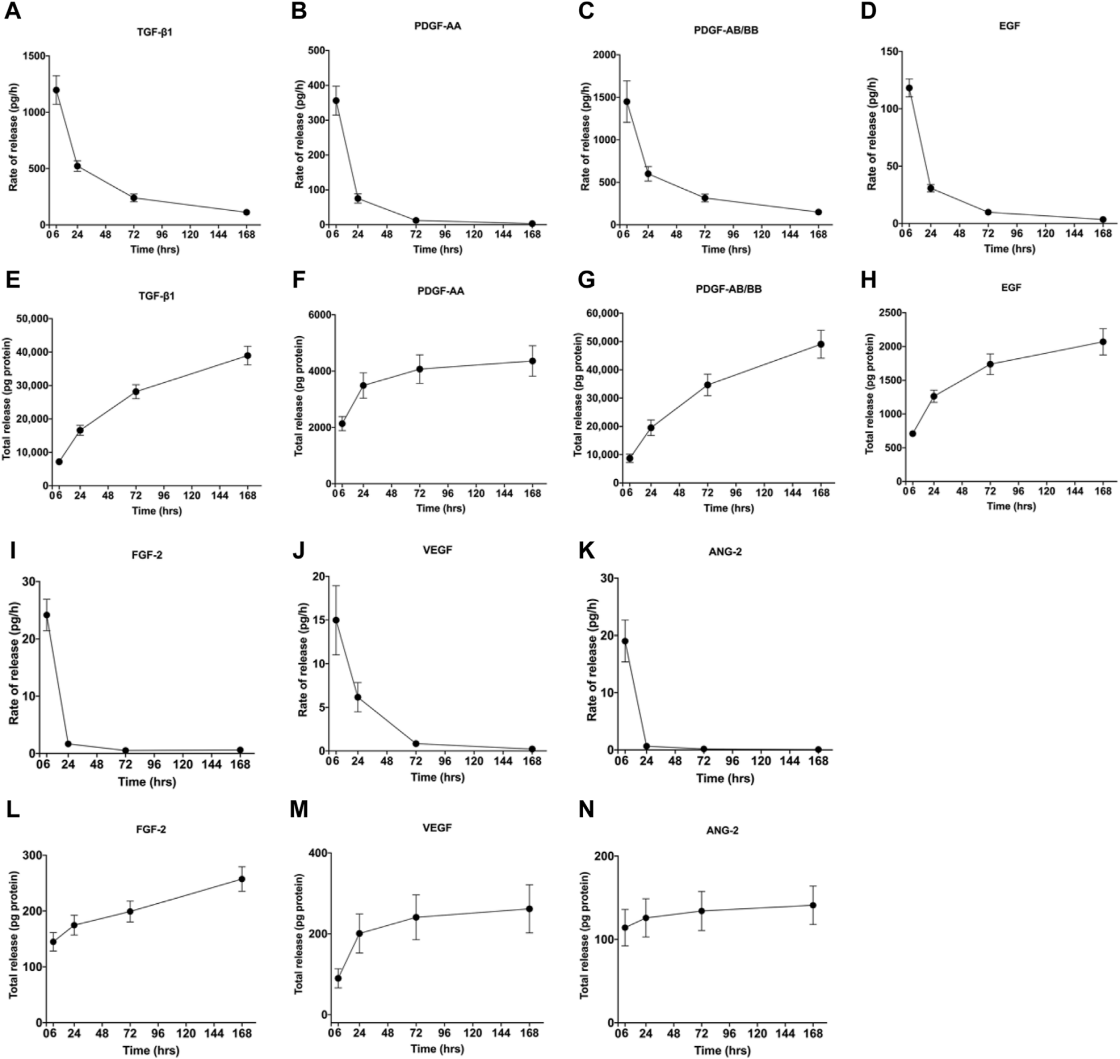
Rate of release and total release of GFs. The figure shows the rate of release and total release of the tested GFs from the L-PRF membrane up to 168 h. Panels **(A, E)** display TGF-β1, **(B, F)** PDGF-AA, **(C, G)** PDGF-AB/BB, **(D, H)** FGF-2, **(I, L)** EGF, **(J, M)** VEGF, and **(K, N)** ANG-2 for rate of release and total release, respectively. Mean ± SD is displayed at each time point. Open access, MDPI.

**FIGURE 4:**
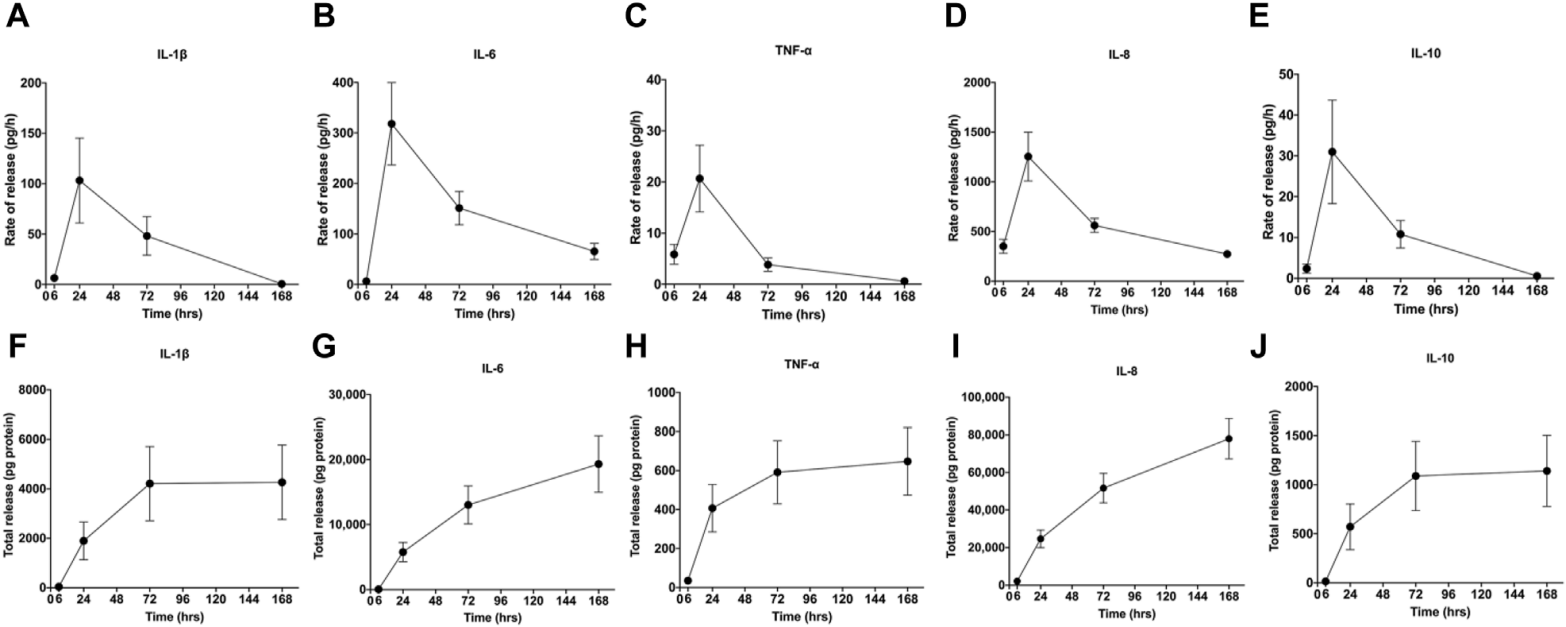
Rate of release and total release of cytokines. The figure shows the rate of release and total release of the tested cytokines from the L-PRF membrane up to 168 h. Panels **(A, F)** display IL-1β, **(B, G)** IL-6, **(C, H)** TNF-α, **(D, I)** IL-8, **(E, J)** and IL-10 for rate of release and total release, respectively. Mean ± SEM are displayed at each time point. Open access, MDPI.

**FIGURE 5:**
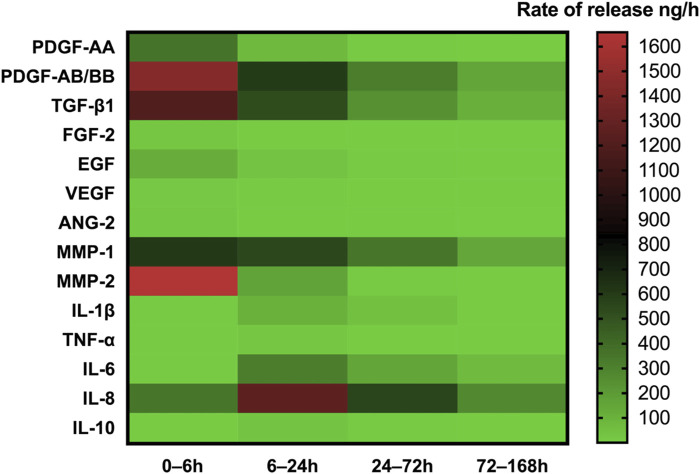
Heatmap of the rate of release of GFs, cytokines, and MMPs up to 168 h. The heatmap represents the changes in the mean of release rate of GFs, cytokines, or MMPs over 168 h. The release rate is indicated according to the color scale representing different levels of rate of release per hour (green—lowest, red—highest rate of release). Open access, MDPI.

During the centrifugal preparation of PRF, activated platelets release many α particles. The cytokines and growth factors known in PRF include platelet-derived growth factors A and B (PDGF-A and B), transforming growth factor β1 (TGF-β1), insulin-like growth factor 1 (IGF-1), VEGF, interleukin-1β (IL-1β), interleukin-4(IL-4), and tumor necrosis factor-alpha (TNF-α) (Figure 2) (Dohan et al., 2006b; a; Lorenzo et al., 2008; Dohan Ehrenfest et al., 2009a). In addition, the total amount of protein released by PRF after 10 days was 9,261.89 ng/mL, and of all the released proteins, PDGF-AA was released in the highest amount, followed by TGF-β 1, PDGF-BB, PDGF-AB, VEGF, EGF, and IGF (Kobayashi et al., 2016). The release rate and duration of growth factors in PRF play an important role in bone regeneration. Xuzhu Wang et al. reported the release kinetics of growth factors, cytokines, and matrix metalloproteinases (MMPs) from PRF at 6, 24, 72, and 168 h. For growth factor and MMPs released from L-PRF, the results showed an exponential decrease in the release rate with time, consistent with a first-order kinetic (Figures 3, 4). In the meantime, a heatmap was generated using the mean of total amounts of release per hour to show the changes in the release rate of growth factors, cytokines, or MMPs over 168 h (Figure 5). For growth factors and MMPs, the highest release was found at the first 6 h, followed by a reduction in concentrations, although with different rates of decrease, while the highest release rates for cytokines and the chemokine occurred from 6 to 24 h (Wang X. et al., 2022). Besides, kinetic analysis of the released factors showed that, in contrast to the bimodal release of TGF-ß1 in L-PRP and clot, in L-PRF, TGF-ß1 showed a single-peak release at day 7; the release profile of IGF-1 in L-PRF showed a gradual release over the first 3 days, and at the same time, in L-PRF, the release of IL-1ß reached the peak of its release at day 1 (Schär et al., 2015). Katharina Zwittnig et al. evaluated the release kinetics of epidermal growth factor (EGF), VEGF, TGF-β1, PDGF-BB, and MMP-9 growth factors in liquid and solid PRF over a ten-day period, and the results showed that EGF was released in the highest amount after 7 h, and the release of VEGF, TGF-β1, PDGF-BB, and MMP-9 reached its highest amount on day 7. The amount reached the highest, and the release of all growth factors decreased significantly on days 7–10. The percentage of the release of five different growth factors in liquid and solid PRF at different time points are shown in Table 2 (Zwittnig et al., 2022). In summary, the growth factors released from PRF can effectively promote angiogenesis and bone regeneration, but the growth factors are released at a faster rate within 24 h, and most of the growth factors have been released within 7 days (Table 2). Therefore, reducing the degradation rate of PRF, slowing down the release of growth factors and prolonging the osteogenesis is one of the urgent problems to be solved.

**TABLE 2:** A table of percentage release of five different growth factors from liquid and solid PRF at different time points.

		Consistency	
		Liquid	Solid
Growth factor	Time point	Percentage of released factors (%)	Percentage of released factors (%)
EGF	1 h	11.68	4.82
	7 h	47.49	27.08
	1 d	72.76	52.17
	2 d	84.59	72.51
	7 d	97.81	95.09
	10 d	100	100
MMP-9	1 h	7.51	10.27
	7 h	20.36	26.38
	1 d	36.06	38.46
	2 d	61.23	52.01
	7 d	93.70	83.32
	10 d	100	100
PDGF-BB	1 h	14.90	26.43
	7 h	24.48	39.71
	1 d	31.06	48.40
	2 d	43.43	56.09
	7 d	80.61	87.29
	10 d	100	100
TFG	1 h	20.89	8.74
	7 h	38.09	16.98
	1 d	50.07	26.44
	2 d	59.74	46.70
	7 d	91.86	84.16
	10 d	100	100
VEGF	1 h	15.94	6.00
	7 h	32.20	16.34
	1 d	49.78	34.49
	2 d	66.12	54.60
	7 d	91.79	90.19
	10 d	100	100

In addition, different platelet derivatives contain different concentrations of growth factors, and a comparison of the growth factor content in PRP, PRF and CGF is shown in Table 3 (Qiao et al., 2017). Meanwhile, Miquel Saumell-Esnaola et al. compared the content of growth factors and total proteins in PRP and pEVs. The results showed that proteins with high expression in PRP, such as c-reactive protein, dipeptidyl peptidase-4, IGFBP-2, IL18-binding protein, thrombospondin-1, VCAM-1, etc., had very low expression in pEVs; platelet factor 4, CCL5, and VDB had almost the same expression in PRP and pEVs samples; and trichothecene factor 3 (TFF3) and PECAM-1 were even more highly expressed in pEVs than in PRP (Saumell-Esnaola et al., 2022).

**TABLE 3:** A table of concentrations of growth factors contained in different platelet derivatives.

	Growth factor levels of different platelet-rich derivatives (ng/mL)		
Growth factors	PRP	PRF	CGF
PDGF-BB	155.20 ± 57.67	146.36 ± 52.31	175.10 ± 57.09
TGF-β1	488.76 ± 240.77	560.81 ± 265.91	584.89 ± 292.50
IGF-1	236.07 ± 222.10	274.36 ± 212.14	321.42 ± 150.30
VEGF	242.29 ± 97.64	259.39 ± 172.79	238.14 ± 149.89
Basic fibroblast growth factor (bFGF)	82.24 ± 64.51	126.86 ± 58.08	130.56 ± 67.66

**FIGURE 6:**
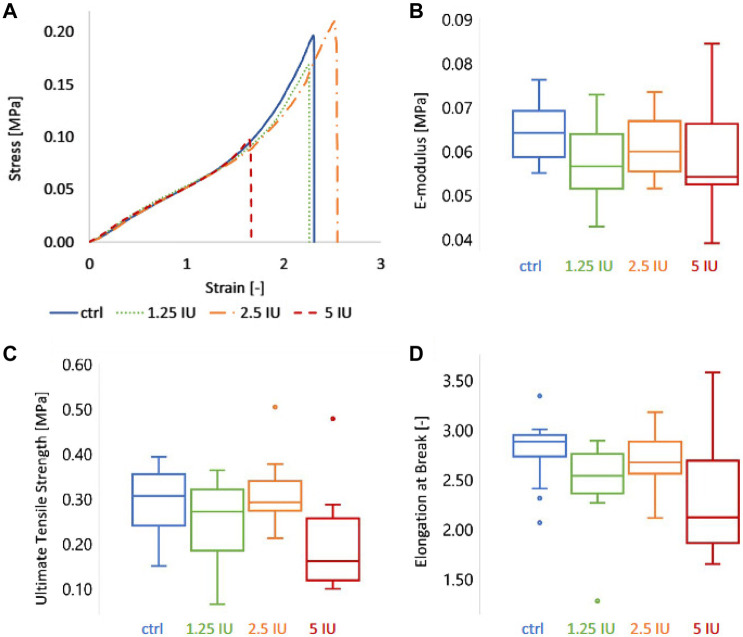
The mechanical characteristics of L-PRF membranes were characterized by tensile testing. Four groups of membranes were compared: ctrl = control (or 0 IU enoxaparin), 1.25 IU, 2.5 IU, and 5 IU enoxaparin-supplemented L-PRF membranes. **(A)** Representative stress-strain curve, **(B)** E-modulus, **(C)** ultimate tensile strength, and **(D)** elongation at break of the four L-PRF groups. Copyright 2020, Wiley.

Bone healing is a dynamic process regulated by both biochemical and biophysical signals and the mechanical properties of bone graft materials are of great significance for bone regeneration. Anna Ockerman’s study evaluated the mechanical properties of L-PRF films and explored the effect of anticoagulation treatment on the structural and mechanical properties of PRF, showing that the mean e-modulus, ultimate tensile strength, and elongation at break of PRF (control group) were 0.07 MPa, 0.29 MPa and 2.78 times, respectively (Figure 6) (Ockerman et al., 2020).

## 4 Mechanism of PRF to promote osteogenesis

Bone regeneration can be a complex process based on the interaction of osteogenic and angiogenic processes and the success of bone regeneration depends on the degree of vascularization of the bone defect area, especially of large scaffolds (Diomede et al., 2020). Adequate blood supply and neovascularization are necessary prerequisites for bone regeneration, especially in the early stages of bone regeneration, not only to provide nutrition and oxygen to the bone defect area but also to support and regulate osteogenic activity at different cellular levels through structural pathways, and paracrine pathways (Grosso et al., 2017; Rather et al., 2019). During bone tissue formation, a variety of cells are often involved, such as mesenchymal stem cells, osteoblasts, and osteoclasts, among which, MSCs can regulate osteogenesis by activating ERK/MAPK, BMP-SMAD, TGF-β, Wnt, and Notch signaling pathways. The bone-forming activity of osteoblasts and the bone-resorbing activity of osteoclasts must be balanced for normal bone remodeling. PRF can regulate the above cells and has certain immunomodulatory and antibacterial effects, which can strongly promote the repair of bone defects.

### 4.1 Effect of PRF on angiogenesis

Angiogenesis is crucial for tissue engineering and bone healing to avoid apoptosis and necrosis of implanted and newly formed tissues (Carmeliet, 2000). Studies have shown that impaired function of the hypoxia-inducible factor-1α/platelet-derived growth factor-β (HIF-1α/PDGF-β) axis may be the primary mechanism of insufficient angiogenesis (Guo et al., 2020). VEGF is a central regulator of angiogenesis, whose overexpression in human bone progenitor cells promotes the vascularization of bone-derived grafts. Meanwhile, during osteogenesis, angiogenesis and osteogenesis are physiologically coupled by VEGF, whose role is related to Notch signaling pathway activation (Burger et al., 2022; Grosso et al., 2023). It shows that i-PRF promotes the expression of VEGF, PDGF, E-selectin, and intercellular adhesion molecule-1 (ICAM-1) in endothelial cells and primary osteoblasts coculture system and promotes angiogenesis (Dohle et al., 2018), whose effects are mainly mediated by hematopoietic stem cells (CD34^+^) and endothelial progenitor cells (CD34+/VEGR-2+/CD133+) *in vivo* (Caloprisco et al., 2010). In addition, VEGF released from platelet-rich fibrin matrix (PRFM) can participate in endothelial cell mitosis via ERK signaling pathway, which effectively induces trabecular endothelial cell proliferation and improves trabecular angiogenesis (Roy et al., 2011). At the same time, L-PRF also significantly enhances the proliferation and migration ability of human umbilical vein endothelial cell (HUVEC) and promotes tube formation through the EGFR signaling pathway (Park et al., 2018; Ratajczak et al., 2018). In addition, PRF prepared by both high (2,700 rpm, 719 g) and medium centrifugal force (1,500 rpm, 222 g) had a vascular regenerative effect; however, the PRF-medium group had a more significant promotion effect. The number of vessels, vessel density, vascularization percentage, and vascularization parameters were higher in the PRF-medium group than in the PRF-high group (Kobayashi et al., 2015; Kubesch et al., 2019).

### 4.2 Effect of PRF on osteogenesis-related cells

#### 4.2.1 The effect of PRF on BMSCs

The abundant platelets and growth factors in PRF recruit stem and progenitor cells to the defect site. At the same time, the three-dimensional scaffold structure provided by its constitutive fibrin network promotes the proliferation and differentiation of the recruited cells to achieve synergistic osteogenesis (Wang et al., 2019; Holly et al., 2021). Studies have shown that BMSCs play a crucial role in bone formation by activating osteogenic-related signaling pathways, such as BMP/SMAD, TGF-β, ERK/MAPK, Wnt, and Notch signaling pathways.

**FIGURE 7:**
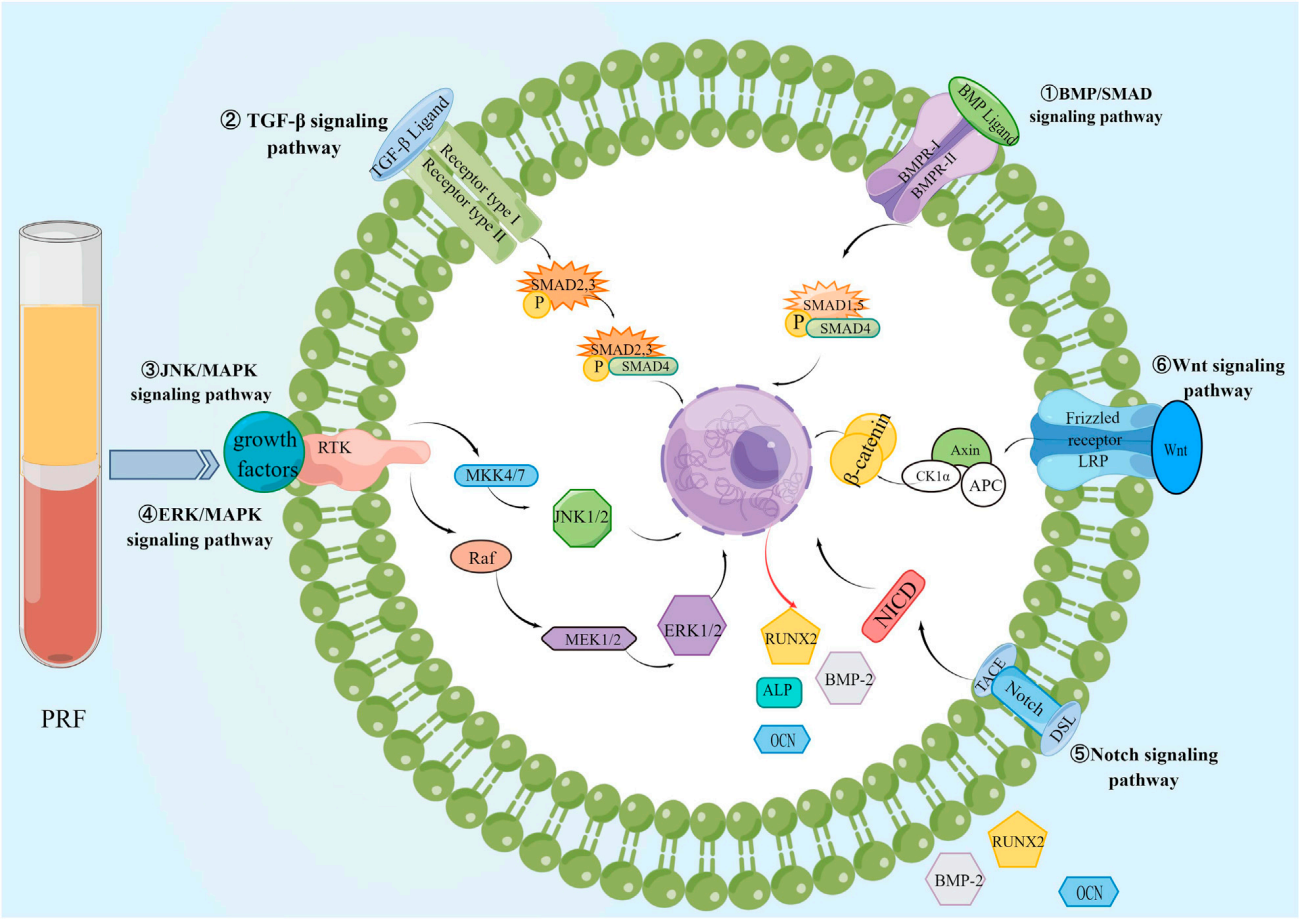
The effect of PRF on BMSCs. PRF promotes bone regeneration by activating BMP/SMAD, TGF-β, ERK/MAPK, JNK/MAPK, Wnt, and Notch signaling pathways in BMSCs.

It is known that Runx2 expression is a marker of osteoblast differentiation and the first and largest precise bone formation marker gene. PRF activates BMP receptors, triggers intracellular SMAD1/5/8 phosphorylation, induces Runx2 gene expression, and promotes osteogenic differentiation of BMSCs through the BMP-2/SMAD signaling pathway (Wu et al., 2022). Recently, a proteomic study showed that PRF lysates also possess intense TGF-β1 activity, which can activate the TGF-β receptor 1 kinase signaling pathway, thereby increasing the expression of BMP-2 and related genes ID1 and ID3. Meanwhile, PRF lysate can lead to phosphorylation and nuclear translocation of Smad1/5 in serum-starved cells. Thus, PRF also has BMP-like activity and activates the BMP-SMAD signaling pathway (Kargarpour et al., 2021c). In addition, application of PRF conditioning medium to SM-Mscs isolated from rabbit maxillary sinus could upregulate the expression of p-ERK1/2 and Runx2, indicating that PRF could accelerate bone healing and improve the quantity and quality of new bone through ERK/MAPK signaling pathway, (Wang J. et al., 2022). Meanwhile, recent studies have shown that PRF promotes the proliferation, migration, and osteogenic differentiation of human bone marrow MSCs by activating the ERK/MAPK pathway. The Western blot showed that the application of ERK inhibitor U0126 reduced PRF-induced expression of p-ERK and Runx2, and alizarin red staining showed that U0126 diminished PRF-induced mineralization of hBMSCs nodule formation (Wang et al., 2023). Meanwhile, PRF and insulin-like growth factor-1 (IGF-1) upregulate RUNX2, osterix (OSX)、osteocalcin (OCN) and ERK expression in human periodontal ligament stem cells (PDLSCs) during different periods of osteogenesis, and promote periodontal ligament stem cell proliferation in the alveolar fossa by activating the mitogen-activated protein kinase (MAPK) signaling pathway (Li X. et al., 2018). During the restoration of alveolar bone defects in rabbits with autologous BMSCs combined with PRF, Notch1, and Wnt3a signaling molecules increased (Zhou et al., 2016), therefore, PRF can also regulate the proliferation, adhesion, and osteogenic differentiation of BMSCs through Notch and Wnt signaling pathways. PRF possesses the capacity to boost the proliferation of human dental pulp stem cells (hDPSC) and stimulate their differentiation into osteogenic or dentinogenic cells through the activation of the Notch signaling pathway (Zhang et al., 2023). Compared to PRP, PRF stimulates osteogenic differentiation of periodontal ligament stem cells, significantly increasing the calcification percentage and accelerating osteoblast mineralization (You et al., 2019; Thanasrisuebwong et al., 2020). The effect of PRF on MSCs is shown in Figure 7.

#### 4.2.2 Effect of PRF on osteoblasts

Osteoblasts, which can synthesize extracellular matrix (ECM), control mineralization, and regulate bone growth, are critical cells in bone tissue regeneration (Brunette and Chehroudi, 1999), and various signaling molecules regulate their proliferation and osteogenic activity. Osteoprotegerin (OPG) and receptor activator of nuclear factor κ B ligand (RANKL)OPG and RANKL are expressed by osteoblasts, with OPG acting as a naturally occurring inhibitor of osteoclast differentiation, and binding to RANKL prevents RANKL from interacting with RANK. Increasing the OPG/RANKL ratio promotes the transformation of osteoblasts to maturation and inhibits the formation of osteoclasts. Studies have shown that PRF can increase the number of OPG secreting cells and the OPG/RANKL ratio, thereby promoting the early differentiation of osteoblasts (Chang et al., 2010). In addition, the ERK/MAPK signaling pathway is the core of the signaling network involved in the regulation of cell growth, development, and differentiation, and plays a crucial role in cell proliferation, differentiation, apoptosis, and autophagy. PRF can activate ERK1/2, phosphorylate substrates in the cytoplasm, or translocate to the nucleus to phosphorylate transcription factors, thereby regulating the expression of target genes. Western blot was used to detect the expression of p-ERK, RANKL and OPG in human osteoblast cell line U2OS cells. The results showed that PRF promoted the phosphorylation of ERK and upregulated the expression of OPG in U2OS cells (Chang et al., 2010). In addition, PRF as a mito-gen promotes adhesion and proliferation of human osteoblast cell line U2OS cells and upregulates the expression of collagen-related proteins, such as phosphorylated Akt, heat shock protein 47 (HSP47) and lysine oxidase (LOX). Among them, Akt, a down-stream effector of the pi3k-dependent signaling cascade, PRF may promote osteoblast proliferation through the Akt signaling pathway. Bone tissue formation may be due to increased synthesis and deposition of HSP47/LOX accumulation, and PRF can upregulate the expression of HSP47 and LOX proteins in human osteoblasts (Wu et al., 2012). Rapid adhesion of osteoblasts on the surface of titanium surfaces is a vital prerequisite for osseointegration and bone healing, and PRF increases the number and length of filamentous pseudopods of osteoblasts, which can reverse the adhesion of osteoblasts on titanium surfaces reduced by zoledronic acid (Steller et al., 2019). To explore whether the osteoinductive effects are the same for different types of PRF, Kostantinos Kosmidis et al. evaluated the impact of A-PRF+, L-PRF, and i-PRF on osteoblast-like cell lines *in vitro*. They showed that all three PRFs enhanced the osteogenic potential of osteoblasts, with A-PRF+ better promoting the functional differentiation of osteoblasts with the highest bone mineralization potential. At the same time, i-PRF focused on inducing early osteogenic differentiation (Kosmidis et al., 2023).

#### 4.2.3 Effect of PRF on osteoclasts

Osteoclasts, as one of the typical cells of bone tissue engineering, play an essential role in the lysis and resorption of bone tissue and contribute to bone maintenance and remodeling. Platelet preparations containing serum components inhibit osteoclast differentiation and promote the proliferation of MG63 cells by upregulating osteogenic differentiation markers of osteoblasts MG63 *in vitro* (Agis et al., 2013). Meanwhile, PRF downregulates the expression levels of osteoclast marker genes TRAP, cathepsin K, dendritic cell-specific transmembrane protein, nuclear factor of activated T cells, and osteoclast-associated receptor, and inhibits the differentiation of bone marrow hematopoietic progenitors to osteoclasts (Kargarpour et al., 2020b; Kargarpour et al., 2021a). The inhibitory effect of PRF on osteoclasts was further enhanced when combined with biphasic calcium phosphate (BCP.) Anil Kumar et al. investigated the inhibitory effect of platelet-rich fibrin/biphasic calcium phosphate on osteoclasts and its mechanism in a periodontitis model. The results showed that PRF/BCP inhibited RANKL-induced osteoclast differentiation of PBMCs. Meanwhile, the apoptosis-specific DNA fragmentation assay showed a typical ladder pattern of inter-ribosomal DNA fragments, indicating that PRF/BCP is an effective inducer of osteoclast apoptosis. In addition, PRF/BCP regulated the expression of apoptosis-related proteins and mRNA in osteoclasts; the term pro-apoptotic protein Bax was upregulated, while the anti-apoptotic proteins Bcl-2 and Bcl-xL were decreased, promoting osteoclast apoptosis *in vitro* through the mitochondrial apoptosis pathway. Furthermore, PRF/BCP inhibits RANKL-induced NF-κB activation, activates caspase-3 and caspase-9 in mature osteoclasts, and promotes apoptosis in osteoclasts (Kumar et al., 2019). PRF/BCP, as a synergistic combination, may serve as an effective inhibitor of osteoclast formation in chronic periodontitis. First, PRF/BCP may effectively prevent osteoclast formation by inhibiting TRAP. Second, PRF/BCP may also inhibit the expression of molecules related to MAPK and NF-κB signaling pathways that may regulate periodontal inflammation and osteoclast formation. Finally, PRF/BCP counteracts osteoclast-associated effects by reducing the expression of osteoclast transcription factors and marker genes c-Fos, NFATc1 and TRAF 6, bone resorption enzymes, histone k, TRAP, and MMP-9 (Kumar et al., 2021).

### 4.3 Effect of PRF on immunomodulatory

During bone healing, immune cells phagocytose and remove microorganisms, necrotic tissues, and temporary fibrin matrix while secreting anti-inflammatory and chemotactic mediators, initiating further recruitment of osteoblasts and MSC, and creating an immune microenvironment conducive to bone regeneration (Zhang et al., 2022). Thus, immunomodulation is crucial for osteogenesis. PRF can play an anti-inflammatory role by decreasing the activity of M1-type macrophages and dendritic cells and promoting the activation of M2-type macrophages. PRF significantly inhibited the expression of M1-type macrophage markers, TNF-α and 1L-6, and inhibited the phosphorylation of p65 and nuclear translocation, which inhibited the activation of M1-type macrophages through the NF-κB signaling pathway in RAW264.7 macrophages. Moreover, PRF promoted the expression of M2 type macrophage markers ARG1 and CD206, activated the TGF-β signaling pathway or increased the activity of the TGF-β signaling pathway to promote M2 type macrophage activation (Zhang et al., 2020; Kargarpour et al., 2021b; Kargarpour et al., 2023). At the same time, PRF promotes the polarization of monocyte-derived macrophages to an “M0/M2-like” phenotype and promotes wound healing by inducing the conversion of CD4^+^ T cells to a regulatory phenotype via glycoprotein A repeat dominant protein (GARP) (Trzeciak et al., 2022). At the transcriptional level, PRF exerted an anti-inflammatory effect by decreasing the expression of IL-1β, NLRP3, CAS11, and IL-18 in LPS-induced macrophages, reducing ROS release in RAW 264.7 cells activated by LPS, and inhibiting macrophage pyroptosis (Sordi et al., 2022). PRF reduced the mRNA levels of the dendritic cell marker gene CD86 and major histocompatibility complex II (MHC II) and inhibited dendritic cell activity. At the same time, hydrogen peroxide is catabolized by peroxidase released from PRF to avoid necrotic cell death due to acute hydrogen peroxide oxidation (Kargarpour et al., 2020a). In addition, activated platelets in platelet-rich fibrin patches produce soluble CD40 ligand (sCD40L), which inhibits the infiltration of CD4^+^CD25+Foxp3+ regulatory T cells (Tregs) into the microenvironment of glioblastomas, and plays an important role in suppressing antitumor immunity (Panek et al., 2019).

### 4.4 PRF antimicrobial-related mechanisms

Bacterial infections have caused about 5% of implant-related procedures to fail in clinical practice (Darouiche, 2004). Thus, the perfect bone regeneration material should promote cell adhesion, proliferation, and differentiation while inhibiting bacterial growth (Kokubo et al., 2003). PRF contains many leukocytes, platelets, antimicrobial peptides, fibronectin, and fibronectin, which have broad-spectrum antibacterial and bactericidal activities (Radek and Gallo, 2007; Moojen et al., 2008; Blair and Flaumenhaft, 2009). Leukocytes, a vital component of the immune system, contain a variety of antimicrobial peptides and enzymes, when stimulated, they degranulate and discharge their contents into phagosomes, killing ingested microorganisms through oxidative and non-oxidative reactions (Levy, 2000). In addition, platelets in PRF play an important role in antimicrobial activity by producing oxygen metabolites, including superoxide, hydrogen peroxide, and hydroxyl radicals, which bind, aggregate, and internalize microorganisms and thus remove pathogens; meanwhile, platelets participate in antibody-dependent cytotoxic processes thereby killing pathogens (Blair and Flaumenhaft, 2009). The cationic peptide CXCL4 released by platelets can bind to hydrophobic bacterial cell membranes and cause permeable death by perforating bacterial cell membranes; therefore, PRF has great promise in treating peri-implantitis (Schuldt et al., 2021). Recent studies have demonstrated that PRF has powerful antibacterial properties against common bacteria in bone tissue engineering, such as *Staphylococcus aureus* and Porphyromonas gingivalis. Furthermore, i-PRF has been found to prevent the formation of *S. aureus* biofilm and could serve as an antimicrobial peptide and bioactive agent to reduce the risk of postoperative infections (Jasmine et al., 2020). Furthermore, PRF includes antimicrobial peptides like human-α-2 macroglobulin that can impede the function of ginkgo proteinase, the primary virulence factor of Porphyromonas gingivalis. PRF secretions also contain hydrogen peroxide and antimicrobial peptides that exhibit antimicrobial properties, hindering the development of Porphyromonas gingivalis (Drago et al., 2013; Rodriguez Sanchez et al., 2021).

## 5 Application of PRF in bone tissue engineering

**FIGURE 8:**
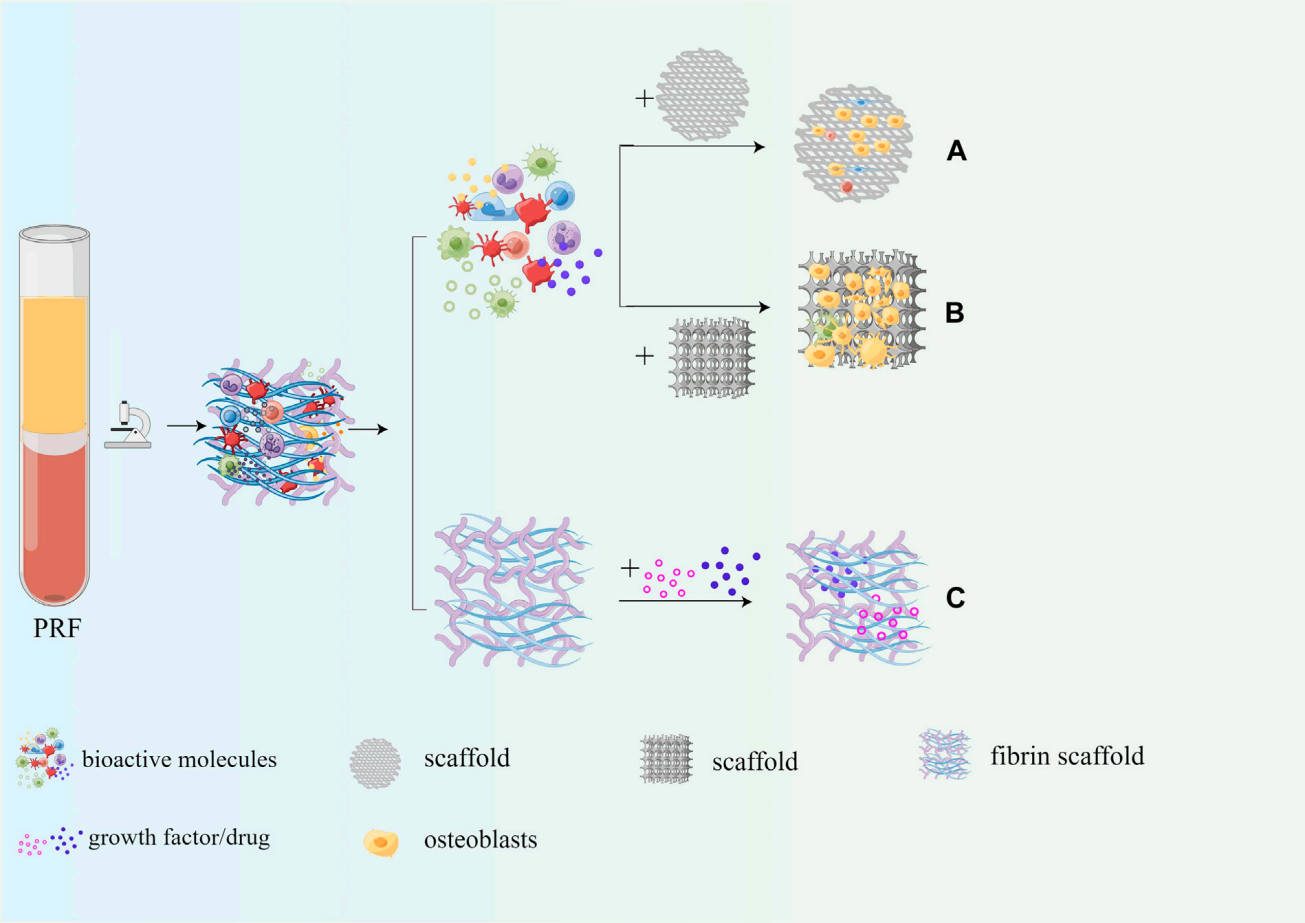
Application of PRF in bone tissue engineering. **(A)** PRF can enhance osteoinduction properties of the scaffold; **(B)** PRF can enhance biocompatibility of the scaffold; **(C)** PRF serves as a drug delivery system.

As mentioned above, PRF contains a large number of autologous growth factors, cytokines, platelets, and leukocytes, which have good osteogenic induction, enhancing adhesion, proliferation, and migration of mesenchymal stem cells and osteoblasts. Therefore, PRF is often combined with common scaffolds for bone tissue engineering to enhance the osteoinductive properties and biocompatibility. Moreover, the fibrin scaffold in PRF can be slowly degraded within 7–14 days without immune rejection. Moreover, PRF can also be used as a drug delivery system to ensure the slow and continuous release of drugs. The application of PRF in bone tissue engineering is shown in Figure 8.

### 5.1 Enhanced osteoinduction properties of the scaffold

Common bone tissue engineering materials, such as nanohydroxyapatite with anti-inflammatory and antibacterial osteogenic potential (Bordea et al., 2020) and zinc silicate/nanohydroxyapatite/collagen scaffolds that promote angiogenesis and bone regeneration (Song et al., 2020), polylactic acid-hydroxyacetic acid (PLGA-BMP-2) scaffolds with sustained release of BMP-2 (Han et al., 2019), possess good osteoconductivity and have great potential for bone regeneration, with the drawback that the scaffolds do not contain autologous cells or growth factors and have poor osteoinductive properties. Notably, autologous growth factors/cytokines at physiological concentrations in PRF can act as bone inducers and are therefore often combined with bone tissue engineering scaffolds as a bioactive material to enhance the osteoinductive properties of the scaffold. MgP_2_Sr-dPRF was prepared by uniformly coating decellularized platelet-rich fibrin (dPRF) on the surface of Sr-doped MgP ceramics, and the results showed that relative to the trabecular bone pattern of MgP_2_S scaffold sections, MgP_2_Sr-dPRF sections showed vascular osteogenic parenchyma containing abundant Haversian canals, bone sinus gaps, and tubular structures. The osteoblasts, osteocytes, and osteoclasts were in reasonable proportions, and the number of new bone formations and maturation was significantly increased in the MgP_2_Sr-dPRF group (Dutta et al., 2022). Furthermore, a recent study prepared a gel-like biomaterial, the L-PRF/PLGA composite graft, by adding platelet-rich fibrin gel to a porous PGLA biodegradable scaffold. It was implanted into sheep mandibular defects, and histological results after 6 weeks showed that the L-PRF-induced group had more bone regeneration and significantly higher bone area occupancy than the non-induced group (Witek et al., 2020). Furthermore, allograft (Bio-Oss^®^), an essential component in guided bone regeneration, was modified with liquid platelet-rich fibrin and BMP-2 to produce an “autograft mimic” (AGM). The platelets and growth factors contained in PRF enhance the osteogenic induction of allogeneic bone grafts, and its fibrous network binds the entire bone replacement material together to form bone grafts. The results showed that AGM promotes bone marrow mesenchymal stem cell proliferation, migration, and mineralization *in vitro* and significantly promotes bone regeneration *in vivo* (Wei et al., 2021). In addition, Lin Zhang et al. successfully prepared a multifunctional three-layer composite scaffold containing platelet-rich fibrin using chitosan/poly (γ-glutamic acid)/hydroxyapatite (CPH) hydrogel prepared by electrostatic action and lyophilization, and applied fresh PRF on the surface of the hydrogel, applied P_2_G_3_ nanofiber film and attached to the surrounding soft tissue. The addition of PRF significantly promoted the osteogenic differentiation of HDPSCs compared to CPH hydrogel, with a significant increase in the formation of osteogenic-related markers and bone-like tissue (Zhang et al., 2019). Multi-walled carbon nanotube/hydroxyapatite (MWCNT/HA) nanocomposite particles that can effectively promote the repair of cylindrical bone defects in sheep humeral epiphysis and femoral epiphysis have good biocompatibility and osteoconductivity, which were mixed with L-PRF in a 1:1 ratio and applied to bone defects, and L-PRF, as a growth factor carrier, significantly improved its osteogenic effect, and more woven bone and lamellar bone was observed in the MWCNT/HA+PRF group (Bastami et al., 2022). Therefore, the combination of PRF and bone tissue engineering scaffold can significantly increase the osteoinductive properties of the scaffold, the expression of osteogenesis-related markers was significantly upregulated, and the quality and quantity of new bone formation were significantly improved.

### 5.2 Enhanced biocompatibility of the scaffold

The biocompatibility of the scaffold itself plays a vital role in the proliferation, migration, and differentiation of osteoblasts and mesenchymal stem cells, and PRF can significantly improve the biocompatibility of the scaffold due to the large number of bioactive components it contains when combined with bone tissue engineering scaffolds. Sarah Al-Maawi et al. used PRF to coat the surface of Pcl-Mesh biochemically, and SEM results showed that a large number of osteoblasts were visible on the surface of Pcl-Mesh covered with PRF, and a large number of cells were present in the lattice gaps of the material. In contrast, when human primary osteoblasts were inoculated on Pcl-Mesh without PRF coating, only a small number of adherent cells could be detected on the grid surface after 7 days (Al-Maawi et al., 2021). Tricalcium phosphate (TCP), which has excellent osteoconductivity properties, was combined with PRF for bio-synergistic bone regeneration. Cells were inoculated onto TCP scaffolds and cultured with PRFM medium. The results showed that PRFM promoted cell proliferation in a time-dependent manner. Both SEM and MTT experiments showed that more actin filaments were visible in the PRF + TCP group, and more osteoblasts extended to the endospores of the TCP bioscaffold. In a rabbit femoral segmental bone defect model, bone regeneration was significantly enhanced in the PRF + TCP group, with the largest collagen-filled area and a more significant number of cells within the collagen (Wong et al., 2021). Chi et al. successfully prepared C/G/DPRF scaffolds by adding DPRF into chitosan-gelatin (C/G) scaffolds. Relative to the C/G and control groups, the OD value of the cells in the C/G/DPRF group was higher and the cell distribution was more homogeneous, while the live-dead staining showed more green fluorescently labeled cells, indicating that there were more live cells (Chi et al., 2019). Yue Song et al. prepared 3D-printed ceramic scaffolds composed of nano-biphasic calcium phosphate (BCP), polyvinyl alcohol (PVA), and platelet-rich fibrin (PRF) using a low-temperature mechanical casting technique. Compared to BCP/PVA scaffolds, BCP/PVA/PRF scaffolds have longer and more abundant pseudopods extending from the surface of BMSCs, a wider area of cell distribution, and a higher cell proliferation rate. BCP/PVA/PRF scaffolds have a better effect on the adhesion, proliferation, and osteogenesis of bone marrow MSCs and can induce more bone formation in a critical rabbit size segmental bone defect model (Song et al., 2018). In conclusion, the addition of PRF significantly enhanced the biocompatibility of the porous scaffolds. More actin filaments could be seen in the cells, and the cells had long and abundant pseudopodia and cells were not only confined to the surface of the material, but also found in the interior of the porous scaffold.

### 5.3 As a drug delivery system

One of the main requirements of a drug delivery system is that the delivered biomolecules/growth factors are released in a controlled manner. With its blood-derived fibrin as a scaffold that naturally degrades in 10–14 days, PRF not only ensures a slow and sustained release of biomolecules/growth factors (Chen L. et al., 2021; Egle et al., 2021; Miron et al., 2021) but also minimizes potential foreign body reactions within the host tissue, in addition, the biomolecules/growth factors carried by PRF can promote vascular and soft and hard tissue regeneration. In bone tissue engineering, antibiotics, non-steroidal anti-inflammatory drugs, and metal nanoparticles (Zalama et al., 2022) are often loaded into PRF fibrin networks to prepare drug-controlled release systems to inhibit local inflammation and infection and improve bone regeneration outcomes. Arita Dubnika et al. prepared a PRF scaffold-based vancomycin (VANKA) controlled release system by first loading VANKA into liposomes or polylactic acid-glycolic acid (PLGA) particles and then loading them into the PRF scaffold. The concentration of VANKA release in the PRF/PLGA_µC_VANKA scaffold was reduced two-fold relative to the PLGA_µC_VANKA scaffold, indicating that PRF inhibited the rapid release of VANKA. This delivery system ensures controlled VANKA release for 6 ∼ 10 days, with the full antimicrobial effect lasting 48 h (Dubnika et al., 2021). Anton Straub et al. collected blood from subjects 10 min after intravenous injection of ampicillin/sulbactam and prepared PRF by centrifugation; the antibiotic concentration in PRF was comparable to the blood concentration, and PRF scaffolds loaded with ampicillin/sulbactam showed significant antibacterial effects against *Haemophilus influenza*, *Staphylococcus aureus*, and *Streptococcus* pneumoniae (Straub et al., 2022). Meanwhile, some investigators loaded clindamycin phosphate (CLP) into PRF fibrin scaffolds, and drug release kinetics showed that approximately 80% of CLP was released from the PRF-CLP scaffold within the first hour after drug loading. Compared with pure CLP solution, CLP combined with PRF showed higher antibacterial activity against *S. aureus* and S. epidermidis with significantly lower minimum bactericidal concentrations (from 1,000 μg/mL to 62 μg/mL). Such a modified PRF-CLP scaffold could provide local antimicrobial activity within the first few hours, reducing the risk of postoperative infection (Egle et al., 2022). Ercan E et al. used T-PRF and collagen loaded with doxorubicin (Doxy), commonly used in periodontitis treatment, and its anti-collagenase significantly inhibits tissue destruction. The pharmacokinetic results showed that the maximum release of collagen/Doxy was only 5.55 ± 0.27 mg/g at 1 h and stopped after 1 h, whereas the T-PRF/Doxy scaffold released 41.72 ± 7.18 mg/g at 1 h and slowly continued to release until 72 h with a cumulative release of 71.98 ± 5.92 mg/g. Compared with the collagen carrier, Doxy loaded in T-PRF was approximately 7 times more (281 mg/g) and had a longer release time with a long-lasting antimicrobial effect (Ercan et al., 2022). Aspirin, a common non-steroidal anti-inflammatory drug, can inhibit TNF- α and IFN-γ production by topical administration, reduce local inflammation and improve cranial repair in rodents and miniature pigs. The PRF/aspirin composite scaffold was prepared by mixing aspirin with blood and centrifuging. The gel-like scaffold structure slowed the aspirin release, and the aspirin concentration rapidly reached 34.3 μg/mL in the first hour, followed by a slow increase. The salicylic acid concentration was 71.2 μg/mL at 48 h and gradually decreased. PRF/aspirin composite scaffolds significantly inhibit inflammation and promote periodontal bone formation (Du et al., 2018). In conclusion, the use of fibrin scaffold in PRF as a drug delivery carrier can inhibit the rapid release of drugs, enhance the antibacterial ability of drugs, and increase their loading, which has great application prospects in the prevention of infection after bone grafting.

## 6 The application of PRF in dentistry

**FIGURE 9:**
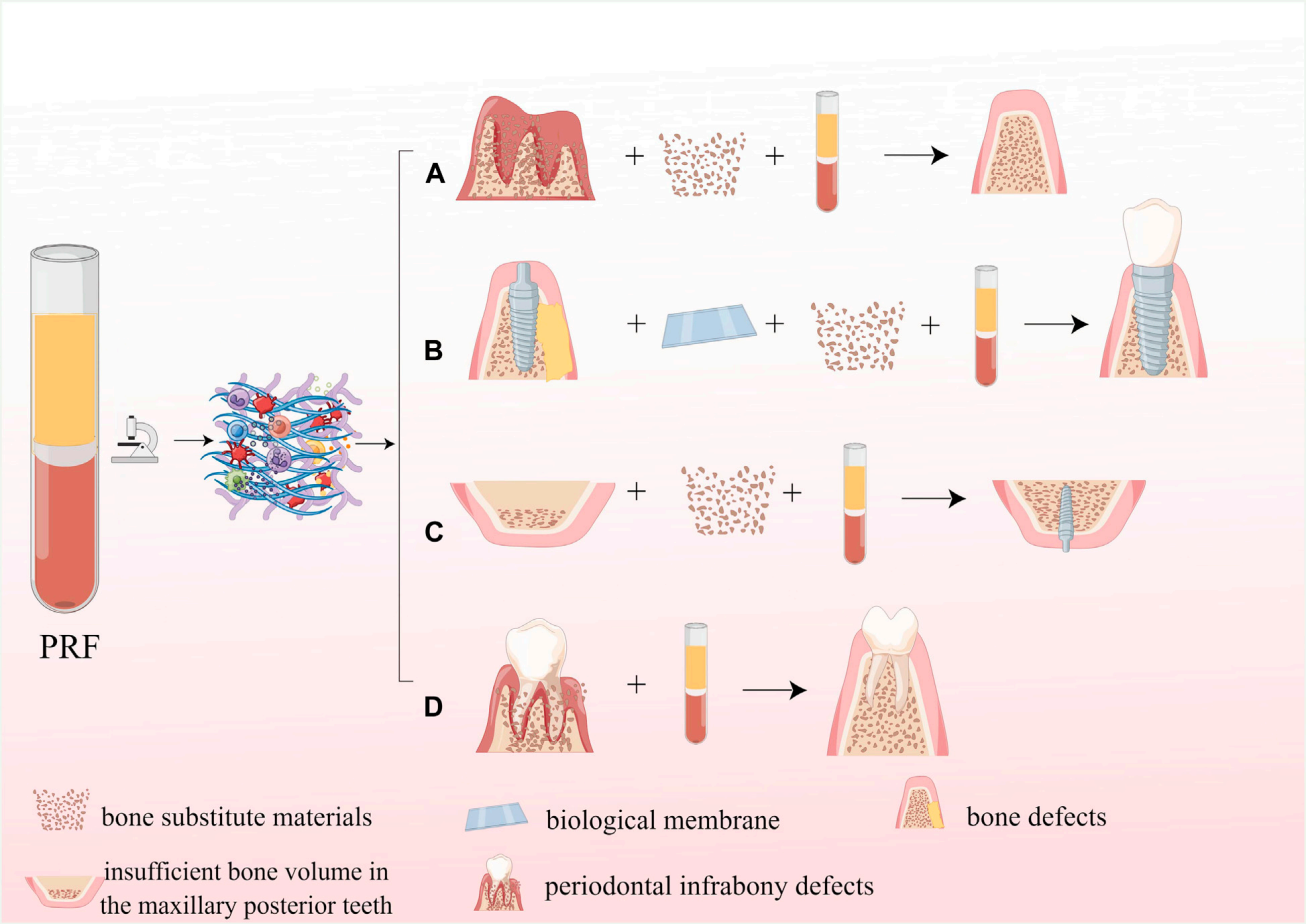
Application of PRF in dentistry. **(A)** The application of PRF in the alveolar ridge preservation; **(B)** The application PRF in guided bone regeneration; **(C)** The application of PRF in maxillary sinus floor elevation; **(D)** The application of PRF for periodontal infrabony defects repair.

Autologous bone grafting is considered the best option for grafting materials; however, its use in clinical procedures is limited due to factors such as higher surgical expenses, the requirement for a secondary surgical site, and the risk of infection. Allograft materials and synthetic BSMs, which imitate the biological properties of natural bone, but only show osseointegration and osteoconductive properties (Zhu et al., 2022), which lack the critical osteoinductive function in the process of bone reconstruction, and suffer from more postoperative resorption, thus limiting their clinical application to some extent. Numerous studies have shown that combining PRF with BSM may be one of the most promising strategies to address the limitations mentioned above of material application in the future, as PRF possesses excellent bone repair-promoting effects due to its high content of growth factors. It was shown that the combination of PRF and BSM promotes the sustained release of growth factors such as VEGF and PDGFA from fibrin scaffolds and promotes angiogenesis in BSM(Yoon et al., 2014; Blatt et al., 2021b), as well as improves the initial viability and metabolic activity of osteoblasts and enhances the early proliferation and migration potential of osteoblasts by increasing the expression of RUNX2, ALP, BMP-2, and OCN(Kyyak et al., 2021). In addition, PRF can promote the expression of IL-6 and TNF-α while upregulating the expression of transmembrane calcitonin receptors and inhibiting the activity of osteoclasts, thus exerting an osteogenic immunomodulatory effect (Blatt et al., 2021a; Park et al., 2023). Biofunctionalization of BSM with PRF can reduce bone resorption and optimize the osteogenic effect of BSMs (Tatullo et al., 2012; Clark et al., 2018). The application of PRF in dentistry is shown in Figure 9.

### 6.1 Application of PRF in guided bone regeneration

Guided bone regeneration (GBR) involves placing a barrier membrane between the soft tissue and the bone defect to create a biological barrier that prevents connective tissue cells and epithelial cells from interfering with bone formation and has a high rate of migration from entering the bone defect area, allowing precursor osteoblasts with potential growth capacity and slower migration rate to preferentially enter the bone defect area and complete bone regeneration without interference (Benic and Hammerle, 2014). As one of the main components of GBR, BSM is often used clinically with PRF. Simone Cortellini et al. mixed granular BSM with chopped L-PRF in a 1:1 ratio, added liquid fibrinogen, prepared as soft block grafts, and then applied it to maxillary bone augmentation. CBCT that is 5–8 months after surgery showed an increase in the bone volume of 4.6 mm, 5.3 mm, and 4.4 mm in the buccolingual direction measured at 2 mm, 6 mm, and 10 mm from the apex of the alveolar ridge, with a mean bone volume increase of 1.05 cm^3^ (Cortellini et al., 2018). Paolo De Angelis et al. performed GBR using a mixture of autologous bone/A-PRF and allograft, respectively, and showed that clinical results were obtained with A-PRF + allograft similar to those obtained with autologous bone + allograft (De Angelis et al., 2022). In another study, PRF was mixed with deproteinized bovine bone mineral (DBBM) and applied to bone augmentation in the severely atrophic alveolar ridge of the maxillary edentulous anterior region. After 1 year, the survival rate of the implants was 98.61%, while the average alveolar bone width was 4.47 mm before the surgery, 9.25 mm right after the surgery, and 7.71 mm 1 year later (Carames et al., 2022). In a study conducted by Gözde Işık et al., they utilized bovine-derived allografts in combination with or without i-PRF for GBR during simultaneous implant placement. The results showed that the bovine-derived allograft + i-PRF group had better buccal bone tissue thickness, less loss of marginal bone level, and lower bone resorption at the 6-month and 2-year postoperative marks (Isik et al., 2021). In conclusion, combining bovine-derived allografts with PRF can significantly improve osteogenesis, better implant stability, higher surgical success rate, and more desirable bone augmentation. In addition, recent studies have shown that platelet-rich fibrin, as a barrier membrane for GBR, has superior osteogenesis than monolayer/bilayer resorbable collagen membranes, with a higher percentage of new bone formation and less fibrosis (Taysi et al., 2018). Although a large number of clinical studies have reported a significant promotion effect of PRF on bone gain, a small number of studies have claimed that the impact of RRF on bone gain remains to be investigated (Hartlev et al., 2019; Hartlev et al., 2020), which may include the following reasons: first, age, gender, physical health status, centrifugal force, rotational speed, and centrifugal time during PRF preparation can affect the quality of the PRF; second, the specific method of PRF application is not straightforward.

### 6.2 Application of PRF in the alveolar ridge preservation

After tooth extraction, the local inflammatory response after surgical trauma, transient upregulation of osteoclasts, and loss of biomechanical stimulation of alveolar bone often lead to disuse atrophy (Avila-Ortiz et al., 2020), and alveolar ridge preservation (ARP) plays a vital role in preventing alveolar ridge resorption after tooth extraction. As mentioned earlier, PRF can inhibit osteoclast production *in vitro*, reduce the expression of osteoclast-associated marker genes, and reduce alveolar bone resorption (Kargarpour et al., 2020b); therefore, PRF is often used clinically for extraction site preservation to reduce alveolar bone resorption and promote bone healing significantly. Warisara Ouyyamwongs et al. used autologous demineralized dental matrix (aDTM) and PRF membrane for extraction site preservation, and the results after 8 weeks of follow-up showed that the aDTM/PRF group had a smaller amount of change in alveolar ridge width and less alveolar bone resorption (Ouyyamwongs et al., 2019). Paolo De Angelis et al. applied L-PRF, allograft bone graft, and allograft of L-PRF mixture were applied separately for ARP and showed at 6 months postoperatively that vertical and horizontal bone resorption was significantly lower in the L-PRF + allograft group than in the allograft alone (De Angelis et al., 2019). In addition, numerous studies have shown that PRF alone applied to extraction site preservation can also increase alveolar bone density, promote new bone formation and improve new bone quality (Ritto et al., 2019). For example, Ana B. Castro1 evaluated the dimensional changes of the alveolar ridge after extraction of multiple teeth in the maxillary anterior aesthetic area with L-PRF or A-PRF+ for ARP versus unassisted alveolar socket healing (control). Histological analysis showed that areas rich in connective tissue fibers (nonmineralized tissue) were seen in the control group samples; in contrast, mineralized tissue in the form of trabeculae was observed in either the L-PRF or A-PRF+ groups and the treatment groups preserved by L-PRF or A-PRF extraction sites showed more bone filling and new bone formation in the alveolar fossa (Castro et al., 2021). Moreover, using PRF-filled extraction sockets can promote soft tissue healing around the extraction sockets while significantly reducing postoperative pain in patients (de Almeida Barros Mourao et al., 2020). In summary, using PRF for extraction site preservation is a promising treatment and should be considered when improved extraction socket healing is required.

### 6.3 Application of PRF in maxillary sinus floor elevation

#### 6.3.1 PRF in combination with BSM for maxillary sinus floor elevation

After the loss of maxillary molars, significant reduction of maxillary bone volume has become one of the common problems in dentistry due to the lack of functional stimulation, pneumatization of the maxillary sinus, and poor bone quality of the maxilla, and maxillary sinus floor elevation as a standard procedure to treat the lack of bone volume in the posterior maxillary region. The commonly used BSM is DBBM, Bio-Oss (Wetzel et al., 1995), which is often mixed with platelet-rich fibrin (PRF) or commercial fibrin (Tisseel) to form a moldable graft material. After conducting preclinical studies on canine maxillary sinus lift, it was found that using PRF in combination with Bio-Oss resulted in a mean osseointegration rate of 43.5% ± 12.4% and a new bone formation rate of 41.8% ± 5.9% at 6 months postoperatively. On the other hand, using Tisseel/Bio-Oss resulted in a mean osseointegration rate of 30.7% ± 7.9% and a new bone formation rate of 31.3% ± 6.4%. Thus, when platelet-rich fibrin was used as an adjuvant to Bio-Oss particles for lateral sinus floor elevation (LSFE), new bone formation at the graft site was significantly increased (Xuan et al., 2014). Based on the guidance of numerous preclinical studies, PRF can be mixed with BSM to fill the maxillary sinus floor, or the PRF membrane can be used as a barrier membrane to cover the buccal bone window. DBBM and L-PRF were used for a bilateral maxillary sinus lift, with DBBM + L-PRF as the experimental group and DBBM transplanted alone as the control group. The results of the histological evaluation indicate that the experimental group had a higher rate of new bone formation (44.58% ± 13.9%) compared to the control group (30.02% ± 8.42%). Additionally, the addition of L-PRF to DBBM allowed for early implant placement (Pichotano et al., 2019). At the same time, another study using PRF mixed with BSM for LSFE with a PRF membrane covering the buccal bone window showed a mean vertical bone height increase of 10.12 mm at 6 months after surgery and no implant loss after a mean follow-up of 43.79 months (Barbu et al., 2018). In conclusion, maxillary sinus floor elevation using a granulated bovine bone substitute and platelet-rich fibrin can be applied as a predictable technique for treating bone height deficiency in the posterior maxillary region.

#### 6.3.2 PRF alone for maxillary sinus floor elevation

As mentioned, PRF has been found to enhance SM-MSC growth, movement, and osteogenic differentiation by activating the ERK1/2 signaling pathway, leading to faster bone regeneration and better quality of newly formed bone. PRF is now being used on its own for transcrestal elevation of the maxillary sinus floor. In a prospective study, Wang Jia et al. showed that the application of PRF as the only bone replacement material for endoscopically assisted transcrestal elevation of the maxillary sinus floor with simultaneous implant placement (PESS) resulted in good clinical outcomes, with a 95.65% implant survival rate at 1 year postoperatively, a mean elevated bone height of 6.72 mm, and an increase in bone density over time in the cervical, middle, and root portions of the implant (Wang et al., 2021). Another randomized controlled clinical study by Huixin Lv et al. similarly showed that vertical bone height increased gradually with time in the PESS group, with a mean bone height increase of 7.67 mm after 18 months of follow-up (Lv et al., 2022). Furthermore, the randomized controlled clinical study by Aida Karagah et al. included ten patients requiring bilateral LSFE, using PRF and L-PRF membranes in one quadrant and FDBA and collagen membranes in the other. According to the findings, both groups experienced a significant increase in the mean ISQ over time. However, the PRF group showed a more significant increase, leading to better implant stability than the other group (Karagah et al., 2022). Platelet-rich derivatives can be used as the sole bone replacement material for maxillary sinus floor elevation. This method has a high success rate for implantation and can achieve the desired bone elevation height. However, it is only suitable for cases where the remaining bone height and density are sufficient to support the initial implant stability.

#### 6.3.3 Application of PRF for schneider membrane perforation

In maxillary sinus floor lift, the most common complication is Schneider’s membrane perforation, which occurs in approximately 20%–25% of cases (Molina et al., 2022), and materials used to repair Schneider’s membrane perforation usually include buccal fat pad, connective tissue, resorbable collagen membrane, fibrous mucosal grafts, and amniotic-chorionic membrane barrier (Holtzclaw, 2015). In studies conducted on animals, it was found that there was no significant difference in healing between collagen membranes and PRF membranes when used to treat perforations in the mucosa of the maxillary sinus, which means that PRF can be used as an alternative to collagen membranes for this purpose (Aricioglu et al., 2017). PRF has a dual function: it not only serves as a physical barrier for repairing perforations but also promotes the healing of bioactive tissue as a “factory.” After PRF repair, intact Schneider’s membrane was visible at the perforation site, and H&E staining showed that the pseudo complexed columnar ciliated epithelium facing the sinus cavity included a lamina propria containing a large number of blood vessels and a deep periosteal-like component, similar to the typical Schneider’s membrane structure; meanwhile, the PRF membrane ensured the formation of new bone at the floor of the maxillary sinus (Xin et al., 2020). Numerous clinical studies have further confirmed the effectiveness of PRF in treating Schneider’s membrane perforations. Esin Kaymaz et al. applied PRF membranes for the repair of Schneider’s membrane perforations with diameters less than 10 mm.6–8 months after surgery, the mean bone height in the non-perforated and perforated groups was 11.18 mm and 10.12 mm, respectively, and a large amount of angiogenesis was observed in both groups, with 100% implant survival and no significant resorption of bone tissue around the implants (Oncu and Kaymaz, 2017). Horia Mihail Barbu et al. used PRF membranes in cases of >15 mm Schneider’s membrane perforation with infection or maxillary sinus mucous cysts. Twelve months after surgery, the mean bone elevation height was 6.43 mm, the histological evaluation showed a mean increment of 52.30% of vital bone, and the mean bone volume/tissue volume (BV/TV) ratio revealed by Micro CT was 50.32% (Barbu et al., 2021). In addition, a recent case report applied PRF membranes together with collagen membranes for a severe Schneider’s membrane perforation, in which five L-PRF membranes were filled with the perforation to ensure closure of the perforation, followed by a collagen membrane covering the PRF to increase the mechanical resistance in the perforated area. Eight months after surgery showed good bone graft healing and bone height recovery (Pinto et al., 2018). In conclusion, PRF can be used as an effective bioactive biomaterial to repair complex and se-vere Schneider’s membrane perforations to provide suitable bone conditions for subsequent implant placement. However, its detailed application method requires further randomized clinical controlled studies.

### 6.4 Application of PRF for periodontal infrabony defects repair

Periodontal infrabony defects are a common factor in tooth loss, and the goal of treatment is to obtain healing of intraosseous defects. Many surgical techniques have been applied to regenerate infrabony defects, including bone grafting, guided bone regeneration, and biological agents (enamel matrix derivatives; growth factors; platelet concentrates) and the combination of these techniques (Figueira et al., 2014), and currently, PRF has been successfully applied to repair intraosseous defects in the periodontal bone. Michele Paolantonio et al. applied PRF with autologous bone graft (ABG) in a randomized, non-inferiority trial for the treatment of periodontally unfavorable bone defects, and clinical and imaging examination at 12 months postoperatively showed that the combination of L-PRF and ABG was not inferior to the combination of enamel matrix derivatives and autologous bone in the treatment of intraosseous defects because significant improvements were obtained in clinical attachment level (CAL), periodontal pocket probing depth (PPD), gingival recession (GR) and defect bone level (DBL) (Paolantonio et al., 2020). A randomized non-inferiority clinical trial by Imena Rexhepi et al. showed that inorganic bovine bone grafts (IBB) combined with L-PRF were not inferior to the combination of IBB with collagen membrane (CM) in the treatment of periodontal infrabony defects, with lower GR and higher DBL gain in the IBB+L-PRF group (Rexhepi et al., 2021). A recent clinical study included twenty patients with chronic periodontitis and bilateral intraosseous defects. The patients were treated with bioactive glass (BG) either with or without PRF for their intraosseous periodontal defects. Combining BG sheets with PRF was more effective in increasing CAL, reducing PPD, and achieving more excellent bone filling with more significant periodontal regeneration than treatment with BG alone (Bodhare et al., 2019). Porous hydroxyapatite (HA) has excellent osteoconductive properties, allowing osteoblasts to grow from the existing bone surface into the adjacent bone material with good clinical results. One investigator applied it in combination with PRF for the treatment of three-wall intraosseous defects in chronic periodontitis and showed that the mean PPD reduction, mean CAL increase, and mean bone filling rate was more significant in the PRF+OFD and PRF+HA+OFD groups than in the open flap debridement (OFD) group (Pradeep et al., 2017). In a word, the application of PRF to treating periodontal bone defects is an effective treatment modality that significantly improves clinical parameters and achieves significant restoration of periodontal bone defects.

### 6.5 Others

Regenerative endodontic procedures (REPs) have been successfully used to treat pulp necrosis in permanent teeth, and their efficacy includes the disappearance of clinical symptoms, periapical healing, increase in root length, thickening of the root canal wall, and apical closure. PRF contains autologous high-density fibrin clots that act as a biological scaffold to support cell proliferation and migration; it also allows an increase in the concentration of local growth factors, stimulates regeneration of diseased pulp tissue, and promotes the formation of dentin and periapical bone tissue. Ahmed Youssef et al. applied PRF to treat adult permanent anterior teeth with pulpal necrosis, and they showed significant improvement in pulp vitality and significant healing of periapical bone tissue at 6-month and 12-month postoperative follow-ups (Youssef et al., 2022). In addition, Le Fort I osteotomy is most commonly used to correct osseous Class III maxillary deformities, and the critical factor for its success is the long-term stability of the bone block. Reza Tabrizi et al. placed PRF at the osteotomy site after bone fixation, and 1-year postoperative lateral cranial radiographs showed that the change (recurrence) in the maxillary A-point relative to the x-axis was 0.45 mm in the PRF group and 1.86 mm in the non-PRF group. The mean value of maxillary change (recurrence) relative to the y-axis was 0.77 mm and 2.25 mm in the PRF and non-PRF groups, respectively; therefore, PRF enhances the stability of the maxilla after Le Fort I osteotomy (Tabrizi et al., 2020). Meanwhile, another investigator used A-PRF and i-PRF mixed with the autologous iliac cancellous bone for the reconstruction of a secondary alveolar cleft in patients with unilateral cleft lip and palate, and their combined application promoted bone formation in the cleft relative to iliac bone graft alone, showing a higher percentage of bone volume and improved periodontal health around the cleft (Dayashankara Rao et al., 2021). At the same time, it has been shown that the application of i-PRF in the treatment of osteoarthritis of the temporomandibular joint and the injection of i-PRF into the joint cavity after arthroplasty can significantly reduce the degree of pain and improve the functional movement of the jaws (Isik et al., 2022). In addition, i-PRF injection based on arthrocentesis can also effectively relieve pain and dysfunction in patients with temporomandibular joint disorders (Karadayi and Gursoytrak, 2021).

## 7 Limitations and optimization of PRF

### 7.1 Limitations of PRF

PRF, as a combination of a scaffold with osteoconductive and a bioactive molecule with osteoinductive ability, has been widely used in bone tissue engineering and clinical practice. However, it has certain limitations, mainly including: first, the poor mechanical properties, lack of rigidity, and rapid degradation of PRF membranes may limit its application in bone augmentation (Sam et al., 2015). Second, although PRF can release growth factors continuously for up to 7–14 days, its duration of action is still much lower than that required for osteogenesis (Kobayashi et al., 2016; Baca-Gonzalez et al., 2022). The growth factor/cytokine release thermogram shows that the release of growth factors is faster at 24 h and slows down over time. The large and rapid release of growth factors in the early phase of PRF may also affect the long-term osteogenic effect (Wang X. et al., 2022). In order to overcome the above limitations, PRF is often optimized, including the following points: to enhance the mechanical properties of PRF and delay degradation to ensure the sustained release of growth factors to improve long-term osteogenesis, which is its main optimization direction.

### 7.2 Optimization of PRF by bone tissue engineering

The mechanical microenvironment of cells is a crucial regulator of cell growth and development (Herberg et al., 2019; McDermott et al., 2019), and the stiffness of the extracellular matrix (ECM) regulates cellular functions such as cell spreading and phenotypic changes in stem and progenitor cells on the planar matrix, which are essential for osteogenic differentiation of stem cells (Wen et al., 2014). However, the gel form of PRF lacks sufficient mechanical strength, and its rapid degradation and burst of growth factor release also conflict with the durable bone regeneration process, which may limit the translational potential of PRF in the clinic. Therefore, PRF is often combined with scaffolds commonly used in bone tissue engineering to enhance its mechanical properties and prolong its duration of action. Recently, some researchers have used coaxial electrostatic spinning technique to prepare PRF-loaded polycaprolactone/chitosan (PCL/CS) core-shell nanofiber scaffolds, which not only possess high mechanical properties but also protect PRF within the core layer from solvent and microenvironmental changes to ensure the continuous release of growth factors. Mechanical properties assessment showed that the tensile strength of PCL/CS-PRF core-shell nanofiber scaffold was 2.98 MPa, which was significantly higher than that of PRF; meanwhile, the results of *in vitro* drug release study showed that the PRF release rate of PCL/CS-PRF nanofiber scaffold was 24.50% after 10 days, indicating a slow and sustained release of PRF from the nanofiber (Rastegar et al., 2021). Composite gels composed of gelatin nanoparticles (GNPs) are effective material for controlled delivery carriers, and a composite hydrogel GNPs+i-PRF was formed by mixing GNPs and i-PRF, which had appropriate mechanical properties. The composite GNPs+i-PRF gel with a concentration of 20 w/v% could achieve yield stress of 33.2 kPa, withstand the weight of an iron strut (weighing 78.26 g) and automatically return to its initial shape in less than 2 min after compression. At 2 weeks postoperatively, the GNPs group produced more vascularity and woven bone in the extraction sockets and lower osteoclast activity, with marked corticalization seen on the alveolar ridge and the highest bone density at the top of the alveolar ridge at 8 weeks postoperatively (Yuan et al., 2021). Similarly, Zhixiang Mu et al. used autologous injectable platelet-rich fibrin (i-PRF) modified with gelatin nanoparticles (GNPs) to develop a double network (DN) hydrogel with mechanical toughness and bioactivity, in which the covalent network of fibrin was used to maintain the integrity of the material. In contrast, the self-assembled colloidal network of GNPs was used to enhance the mechanical properties of the scaffold. Mechanical property evaluation showed that DN hydrogels exhibited a linear elastic followed by brittle fracture mode under compressive loading; the fracture energy of DN hydrogels was significantly higher at 9.2 ± 2.2 kJ m^−3^ compared to the cured iPRF (0.5 ± 0.1 kJ m^−3^). The tensile test results showed that the DN hydrogel exhibited a linear stress-strain curve, a purely elastic response. The mechanical properties of the cured i-PRF gel were weaker, with E of 1.5 ± 0.3 kPa and σt of 1.2 ± 0.2 kPa; the E and σt of DN hydrogel were 14.1 ± 3.1 kPa and 2.5 ± 0.2 kPa, respectively, which were more elastic than i-PRF. DN hydrogel could adapt to the irregular shape of the defect, withstand the pressure developed in the maxillary sinus during animal respiration, and retard the release of bioactive growth factors in i-PRF. i-PRF contributes to early vascularization and bone production in the sinus cavity and provides a valuable solution for treating highly complex localized bone defects (Mu et al., 2020). Sicong Ren and a team of researchers have developed an impressive solution for bone regeneration called PDA@SiO2-PRF. This hydrogel, made from blood-derived proteins PRF, polydopamine (PDA), and SiO2 nanofibers, has a remarkable stiffness that is perfect for maintaining space in bone defects. Additionally, the PDA component in PDA@SiO2-PRF helps prevent the rapid degradation of PRF and provides a sustained ability to promote the growth of new bone tissue. In contrast, the velocity of PDA@SiO2-PRF hydrogel was significantly slower than that of pure PRF hydrogel, and the release of growth factors from PRF was synchronized with its degradation. In addition, due to the rapid degradation of growth factors, the release of growth factors could only be detected within 7 days in the PRF group. In contrast, the release of growth factors in the PDA@SiO2 PRF hydrogel could be observed over 15 days. What’s more the structure of PDA@SiO2-PRF is similar to bone extracellular matrix (ECM) and stimulates the differentiation of bone cells through the yes-associated protein (YAP) signaling pathway (Ren et al., 2022). Combining chitosan (CS) and hydroxyapatite (HAP) is famous for bone scaffolds (Lebourg et al., 2010; Guda et al., 2013; Intini et al., 2018), by mixing novel chitosan (CS)-hydroxyapatite (HAP) with lyophilized platelet-rich fibrin (Ly-PRF) through low-temperature 3D printing technology, the L-PRF-CS-HAP composite scaffold was prepared. Notably, the 2.5% P-C-H scaffold displayed the feeblest compressive strength and modulus, measuring (552.5 ± 21.5) kPa and (9.8 ± 1.9) MPa, respectively. The CS-HAP scaffold exhibited high compressive strength and modulus, 994.5 ± 82.9 kPa and 22.1 ± 4.5 MPa, respectively. With the addition of L-PRF, the compressive properties tended to decrease, and the 2.5% P-C-H scaffold had the lowest compressive strength and modulus, which were 552.5 ± 21.5 kPa and 9.8 ± 1.9 MPa, respectively, but still achieved cancellous bone strength (compressive modulus of about 2–20 MPa). Combining L-PRF with CS-HAP enhanced its mechanical properties while prolonging the release of growth factors, which were still detectable after 35 d of slow release (Sui et al., 2023).

## 8 Prospect

PRF, as a classical bioactive material of autologous origin, has been widely used in bone tissue engineering and dentistry. PRF contains a variety of growth factors, such as TGF-β, PDGF, and VEGF, which can effectively promote vascular regeneration; in addition, it can promote bone tissue repair by promoting the proliferation, migration, and differentiation of mesenchymal stem cells and osteoblasts and inhibiting the activity of osteoclasts; at the same time, it can inhibit inflammation in early osteogenesis to promote bone formation; the leukocytes and antimicrobial peptides contained in its fibrin scaffold can improve local immunity and reduce the risk of infection after bone augmentation surgery. In bone tissue engineering, PRF can be used as a bioactive material to enhance scaffolds’ osteoinductive properties and biocompatibility, and its fibrin scaffold structure can be used as a successful drug delivery system. In dentistry, PRF alone/in combination with bone replacement materials have been successfully applied in GBR, ARP, maxillary sinus floor elevation, periodontal bone defect regeneration, pulp regeneration, and orthognathic surgery. In order to enhance the clinical translation potential of PRF, it is often combined with scaffolds commonly used in bone tissue engineering to enhance its mechanical properties and delay degradation to ensure a continuous slow release of cytokines and improve long-term osteogenesis. However, the current research on PRF is still insufficient, mainly in the following points:

First of all, as far as the PRF preparation method is concerned, the amount of centrifugal force, tube-rotor angle, rotor radius size, and composition of the centrifuge tube during PRF preparation (Miron et al., 2020) can affect fibrin clot formation and structure, and there is no clear implementation standard for the PRF preparation process. Therefore, optimization of various centrifugation equipment is needed in the future, as well as further exploration of the optimal centrifugal force and centrifugation time to further optimize the structure of PRF.

Next, in terms of the composition and biological properties of PRF: First, leukocytes, as an essential immune component, have some potential application in the prevention of postoperative infection in bone augmentation. It has been shown that GFs released from L-PRF may activate cells in the wound and promote more GFs production by host cells, and this induction is mainly attributed to leukocytes within L-PRF. However, some studies have shown that leukocytes significantly increase the secretion of pro-inflammatory cytokines, which may negatively affect the synthesis of type I collagen and ALP by osteoblasts (Baca-Gonzalez et al., 2022). Therefore, further studies are needed to determine the role of leukocytes in bone regeneration. Second, hemoglobin, one of the main components of red blood cells, can restore cell proliferation by iron supplementation, and its lysates can inhibit osteoclast formation (Kargarpour et al., 2021a). Therefore, future studies may consider the beneficial effects of erythrocytes on tissue regeneration without compromising the integrity of the membrane.

Finally, when using PRF as a topical carrier for bone tissue engineering, it is essential to consider drug loading timing carefully. Adding the drug before centrifugation can harm the structure of PRF while adding the drug after centrifugation is optimal as it maintains the fibrin structure and does not disrupt the coagulation cascade (Ercan et al., 2022). It is also vital to preserve growth factors’ biological activity and conformational stability to promote tissue regeneration. Nonetheless, further research is necessary to determine how much the fibrin network in PRF can maintain the biological activity of diverse biomolecules. Besides, in terms of dentistry applications, PRF can increase the secretion of neurotrophic factor (NGF, GDNF) in Schwann cells, which can be used for nerve injury repair. Huang et al. (2020) successfully bridged a 5 mm long sciatic nerve in nude mice using a novel platelet fibrin membrane nerve conduit. The clinical study by Tabrizi et al. (2018) demonstrated that PRF can promote the recovery of neurosensory abnormalities after sagittal osteotomy. However, clinical studies on PRF repair of neurogenic injury are insufficient, and further studies are needed to broaden its clinical application.
